# Skeletal muscle gene expression dysregulation in long-term spaceflights and aging is clock-dependent

**DOI:** 10.1038/s41526-023-00273-4

**Published:** 2023-04-03

**Authors:** Deeksha Malhan, Müge Yalçin, Britt Schoenrock, Dieter Blottner, Angela Relógio

**Affiliations:** 1 Institute for Theoretical Biology (ITB), Charité-Universitätsmedizin Berlin, Corporate Member of Freie Universität Berlin, Humboldt-Universität zu Berlin, and Berlin Institute of Health, Berlin, 10117 Germany; 2 Molecular Cancer Research Center (MKFZ), Medical Department of Hematology, Oncology, and Tumour Immunology, Charité-Universitätsmedizin Berlin, Corporate Member of Freie Universität Berlin, Humboldt-Universität zu Berlin, and Berlin Institute of Health, Berlin, 10117 Germany; 3 Institute for Systems Medicine and Faculty of Human Medicine, MSH Medical School Hamburg, Hamburg, 20457 Germany; 4 Institute of Integrative Neuroanatomy, Charité-Universitätsmedizin Berlin, Corporate Member of Freie Universität Berlin, Humboldt-Universität zu Berlin, and Berlin Institute of Health, Berlin, 10117 Germany; 5 Neuromuscular System and Neuromuscular Signaling, Berlin Center of Space Medicine & Extreme Environments, Berlin, 10115 Germany

**Keywords:** Computational biology and bioinformatics, Systems biology

## Abstract

The circadian clock regulates cellular and molecular processes in mammals across all tissues including skeletal muscle, one of the largest organs in the human body. Dysregulated circadian rhythms are characteristic of aging and crewed spaceflight, associated with, for example, musculoskeletal atrophy. Molecular insights into spaceflight-related alterations of circadian regulation in skeletal muscle are still missing. Here, we investigated potential functional consequences of clock disruptions on skeletal muscle using published omics datasets obtained from spaceflights and other clock-altering, external (fasting and exercise), or internal (aging) conditions on Earth. Our analysis identified alterations of the clock network and skeletal muscle-associated pathways, as a result of spaceflight duration in mice, which resembles aging-related gene expression changes observed in humans on Earth (e.g., *ATF4* downregulation, associated with muscle atrophy). Furthermore, according to our results, external factors such as exercise or fasting lead to molecular changes in the core-clock network, which may compensate for the circadian disruption observed during spaceflights. Thus, maintaining circadian functioning is crucial to ameliorate unphysiological alterations and musculoskeletal atrophy reported among astronauts.

## Introduction

Skeletal muscle is one of the most dynamic and plastic organ of the human body^1^. Skeletal muscle mass is regulated by the dynamic control of protein synthesis and degradation that are sensitive to factors such as nutritional status and physical activity^2^. Aging results in a gradual loss of muscle function, which varies based on sex and the level of muscle activity^3^. The accelerated loss of muscle mass and function, associated with increased adverse outcomes including falls and frailty, results in sarcopenia^4^ (also called muscle wasting).

In mammals, including humans, more than 2300 genes related to the striated muscle (heart and skeletal) are expressed with a circadian pattern^5^. Such circadian (about 24 h) rhythms are the result of the regulation via the endogenous biological clock, known as the circadian clock. The mammalian circadian clock regulates the timing of various physiological processes such as metabolism^6^, myogenesis^7^, and immune system^8^. Data from the circadian gene expression atlas for mammals unveiled different temporal organization between central and peripheral tissues^9,10^. Body temperature rhythms across the day for example are obvious signatures of plasticity and adaptation of mammalian circadian oscillators in peripheral tissues^11^.

The circadian system consists of a central pacemaker the suprachiasmatic nucleus (SCN), located in the anterior hypothalamus of the brain, and peripheral oscillators, present in peripheral tissues such as the liver^12^. The SCN clock synchronizes peripheral clocks by neuronal and hormonal signals to ensure coordinated physiological activities^13^. To remain aligned with the external environment, the clock must be daily re-adjusted, which can be achieved via external time cues known as Zeitgebers^14^. Light is the dominant environmental cue for the SCN and light therapy is reported to improve the sleep quality among patients with neurodegenerative disorders^15,16^, to decrease fatigue symptoms among cancer patients^17,18^, and to improve circadian misalignment in Space^19,20^. Besides light, the circadian system is also sensitive to other Zeitgebers related to meal timing (e.g., fasting and caloric restriction^21^) or to physical exercise^22–24^. At the molecular level, circadian rhythms in gene and protein expression are generated via transcriptional-translational feedback loops (TTFL) where Brain and muscle ARNTL-like 1 (BMAL1; also known as ARNTL) forms a heterodimer with Circadian locomotor output cycles kaput (CLOCK) and regulates the transcriptional activation of Period 1/2/3 (*PER 1/2/3*), and Cryptochrome 1/2 (*CRY 1/2*), which in turn inhibit BMAL1-CLOCK mediated transcription^25^. A further fine-tuning of *BMAL1* is achieved via the opposing effects of RAR-related orphan receptor A/B/C (RORA/RORB/RORC) and Nuclear Receptor Subfamily 1 Group D Member 1/2 (NR1D1/NR1D2)^26,27^.

Recent evidence points towards an important role of the circadian clock in skeletal muscle homeostasis. Circadian rhythms play a prominent role in the maintenance of physiological muscle mass, and the loss of *Bmal1* in a mouse model resulted in severe sarcopenia^28^ with profound consequences for life quality^29^. Aging directly influences circadian parameters leading to a decrease in amplitude and phase shift of the oscillations^30,31^. A mouse model of aging showed defects in synchronization to light-dark cycles^32,33^. Besides aging, studies on the effects of prolonged immobility (bedrest) among individuals due to injury or illness^34^, and in astronauts exposed to microgravity during spaceflight also reported severe muscle loss^35^. Moreover, astronauts exhibited a delay in circadian phases of core body temperature (+1 °C inflight) during their stay at the International Space Station (ISS)^36^, which was also seen in healthy participants during long-term bedrest^37^, as analogue to spaceflight.

Disrupted synchrony between environmental and endogenous circadian cycles during life may reduce workforce health^38^, lead to disease^39^ and decrease lifespan dramatically^40^. Thus, a proper functioning of the circadian clock is critical to maintain homeostasis and physiological functions, and the perturbation of this system may result in severe pathologies including cancer^41,42^. Abnormal changes in circadian rhythms have been reported in jet lag^43^, night-shift workers^44^, cancer^45,46^, metabolic disorders^47,48^, mental disorders^49,50^, and also as a consequence of spaceflight^51–53^. Space travellers report circadian rhythm disruption during spaceflight, which is visible in many physiological aspects, for example shorter sleep durations^54^. Astronauts consciousness and alert reactivity are compromised by alterations of their endogenous circadian rhythms leading to lower performance and associated risk during mission duties^53^. Moreover, circadian misalignment can affect not only sleep, but also medication intake during Space missions^55,56^.

In recent years, studies based on transcriptomics data reported altered circadian signalling pathways due to spaceflight in different peripheral tissues of murine models^51,52,57^. However, a detailed molecular characterization of circadian clock alterations in skeletal muscle due to spaceflight is still lacking. Whether exercise or fasting, based on the time of day, can be used as a preventative measure against spaceflight induced skeletal muscle loss also remains elusive. Physical exercise based on the time of day influences gene expression profiles in skeletal muscle^58^, and fasting was reported to promote a deep quiescent state in muscle stem cells^59^. Physical exercise is already implemented as a countermeasure against physiological alterations under microgravity as reported in spaceflight and bedrest conditions (reviewed in^60,61^). Likewise, also time-restricted feeding improved cardiac glucose usage and ameliorated cardiac dysfunction induced by simulated microgravity in rats^62^. Moreover, different nutritional interventions were tested to trigger muscle metabolism and homeostasis in astronauts^63^.

In this study, we carried out a systematic analysis to characterize the potential functional consequences of circadian clock disruptions in skeletal muscle due to intrinsic (e.g., alterations of the circadian clock due to genetic manipulation of clock genes or aging) and extrinsic factors (e.g., alteration of circadian rhythms in extreme environments like spaceflights and similar artificially generated conditions). For our analysis, we used datasets from 28 published genomics (Microarray, RNA-seq) and proteomics (LC-MS) based studies. To pinpoint the specific alterations in skeletal muscle due to spaceflight, we characterized the circadian phenotype within the skeletal muscle of healthy mammalian tissues, investigated the impact of core-clock specific alterations (*Bmal1* knockout or *Clock* mutant or *CLOCK* knockdown vs. WT) on skeletal muscle phenotype.

In addition, we investigated the impact of spaceflight in alterations of the core-clock network and skeletal muscle-associated pathways, and whether such alterations could be mimicked under bedrest conditions, as an analogue to spaceflight, and inversely, whether hypergravity resulted in opposite changes in the core-clock network compared to microgravity. We compared molecular alterations observed under extreme environments with aging-related alterations in the expression profile of core-clock genes, to investigate whether spaceflights may confer an aging related phenotype to astronauts. Finally, we investigated whether external Zeitgebers like fasting and exercise have an impact on gene expression of core-clock network elements, and in the circadian profiles of skeletal muscle-related genes.

Our study shows that long-term spaceflight results in severe molecular alterations in the expression of circadian clock genes and skeletal muscle-associated pathways, and points to a common molecular dysregulation among these pathways between long-term spaceflight and aging condition datasets. Several core-clock genes like *Rorc/RORC* were downregulated in spaceflight and aging condition datasets, while its expression was upregulated in a fasting condition dataset. In addition, also skeletal muscle-associated genes like muscle atrophy associated gene activating transcription factor 4 (*Atf4/ATF4*) was downregulated in both spaceflight and aging datasets, while its expression in fasting and exercise conditions (depending on the type of exercise) was upregulated. Hence, insights from our study will help us to better understand how to ameliorate and/or reverse musculoskeletal atrophy common among astronauts due to spaceflight. There is an urgent need to establish preventive measures for astronauts using circadian biology to minimize the effect of Space missions on their health and performance^64^. The usage of extrinsic or intrinsic factors may allow to re-establish a robust circadian profile for improved adaptation to microgravity, health and performance on orbit. This can be applicable to, for example, in future extended missions like Deep Space Exploration programs (Moon, Mars), but also during recovery after return to Earth.

## Results

### A topology of circadian gene expression regulation in the mammalian skeletal muscle

**Fig. 1:**
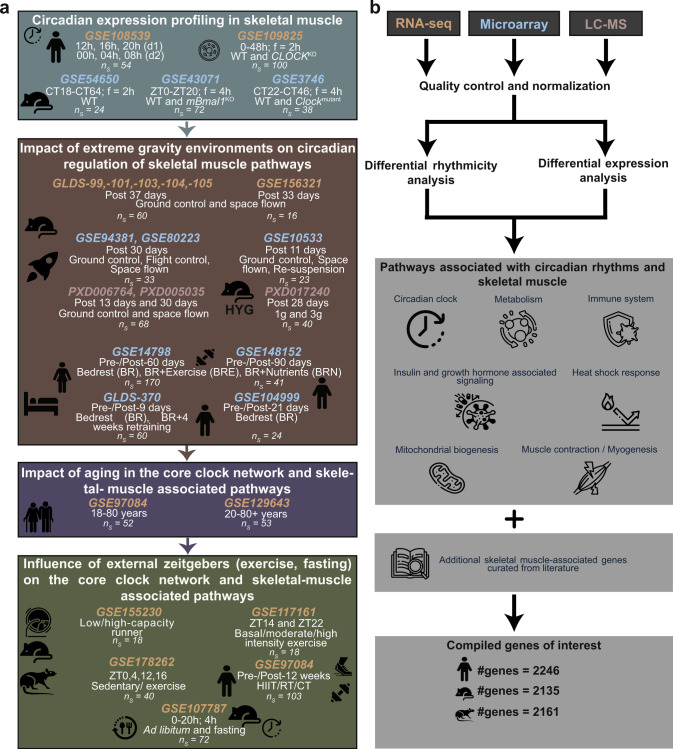
Schematic representation of the analysis workflow to elucidate the regulatory role of circadian rhythms under extreme gravity environmental conditions. **a** Published sequencing (microarray, RNA-seq, LC-MS) datasets based on skeletal muscle were gathered from open source repositories (n_s_ = number of samples; f: frequency). **b** Differential rhythmicity (for circadian datasets) and differential expression analysis were carried out after pre-processing of the raw sequencing datasets. Genes of interest were retrieved from KEGG, Reactome, and literature curation related to skeletal muscle-associated pathways and clock-controlled genes.

Like most mammalian organs, also skeletal muscle exhibits circadian rhythms in gene expression resulting in oscillations in metabolism, and myogenic capacity^7^. To pinpoint the specific molecular changes in mammalian skeletal muscle due to spaceflight, and whether these changes are similar to aging on Earth or can be altered via exercise/food regime, we gathered published omics datasets (microarray, RNA-seq, LC-MS) obtained from skeletal muscle tissue or cells (Fig. 1a), as indicated in the Methods.

Skeletal muscle mass is controlled by multiple signalling pathways such as mTOR signalling, Calcineurin and NFATs^65^. Therefore, to examine the changes in skeletal muscle phenotype, we focused on the gene sets involved in these signalling pathways. The list of genes involved in skeletal muscle-associated pathways were retrieved from KEGG^66^, Reactome^67^, and literature curation^68,69^ (Fig. 1b; Supplementary Dataset 2–4). To prevent discrepancies in the downstream analysis, we retrieved the raw sequencing files and processed all files using the same pipeline and the same statistical cut-offs (Fig. 1b).

**Fig. 2:**
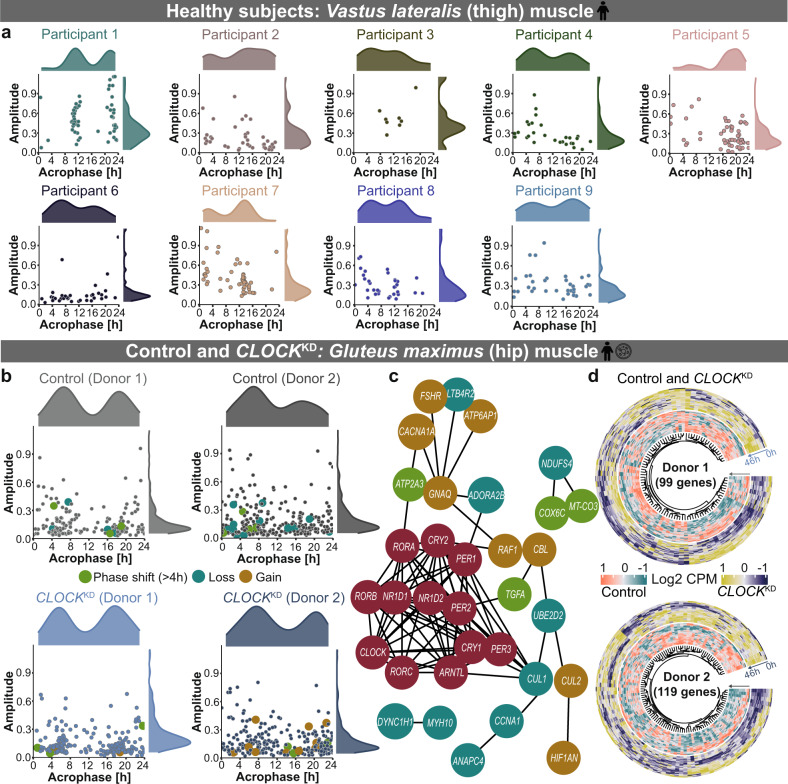
Genes of interest exhibit circadian properties in human healthy muscle tissue and cells which are altered upon *CLOCK*^KD^. Scatter plot depicts the distribution of circadian (~24 h) genes of interest in (**a**) muscle biopsies collected from nine healthy participants and (**b**) myotubes cultured cells from two healthy control donors (upper panel) and siRNA transfected *CLOCK*^KD^ cells (lower panel). **c** STRING network depicts the interaction between differential rhythmic genes due to *CLOCK*^KD^ and the core-clock genes (red circles). **d** Circular heatmap visualizes the differentially expressed genes of interest due to *CLOCK*^KD^ vs. WT in donor 1 (upper panel) and donor 2 (lower panel).

**Fig. 3:**
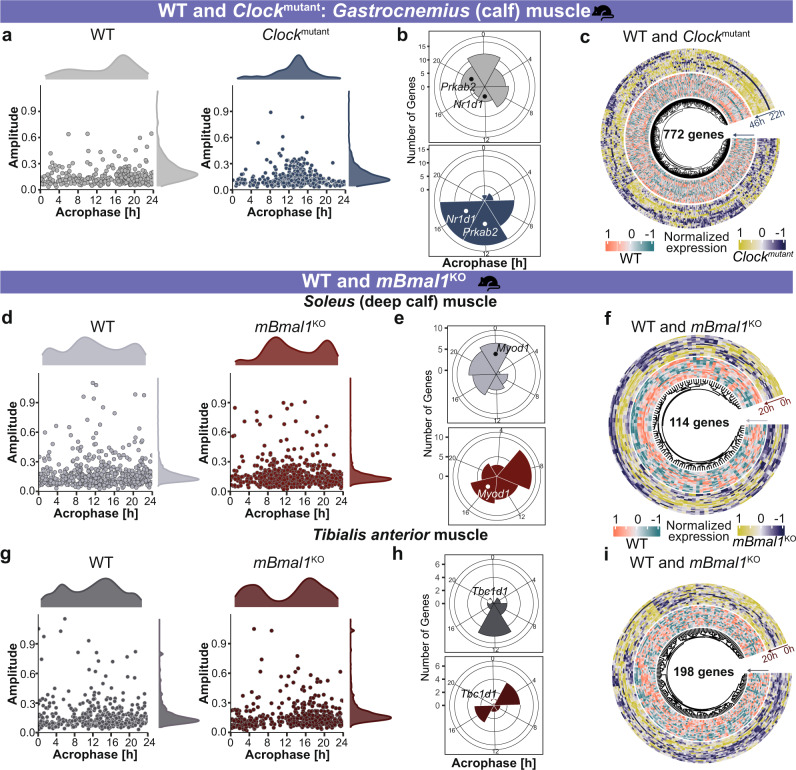
Clock alterations resulted in changes among circadian properties and differential expression in mouse skeletal muscle. **a** Scatter plot depicts the overall distribution of ~24 h genes of interest in WT and *Clock*^mutant^ mice. **b** Acrophase bin plot depicts the distribution of phases of differentially rhythmic genes of interest between WT and *Clock*^mutant^. **c** Circular heatmap plot represents the expression profile of 772 differentially expressed genes of interest between WT and *Clock*^mutant^. **d**, **g** Scatter plot depicts the overall distribution of ~24 h genes of interest in *Soleus* and *Tibialis anterior* muscle of WT and *mBmal1*^KO^ mice. **e**, **h** Acrophase bin plot depicts the distribution of phases of differentially rhythmic genes of interest between WT and *mBmal1*^KO^. **f**, **i** Circular heatmap plot represents the expression profile of 114 (in *Soleus*) and 198 (in *Tibialis anterior*) differentially expressed genes of interest between WT and *mBmal1*^KO^.

To investigate the relevance of circadian regulation on skeletal muscle specific pathways, we evaluated the circadian (~24 h) gene profile in human and mouse time course datasets (Figs. 2, 3). The assessment of circadian gene expression in human skeletal muscle was carried out using RNA-seq data derived from serial biopsies of nine healthy participants (8 males and 1 female; age = 22–54 years) taken every 4 h across two days (Fig. 2a). The circadian phenotype of genes of interest showed individual specific variations in all nine healthy participants (Fig. 2a). In total 335 genes were ~24 h in at least one of the nine participants. These included the myonuclei related gene, Sad1 and UNC84 Domain Containing 2 (*SUN2*), which is essential for the maintenance of muscle mass and the core-clock gene *NR1D1*, known to be relevant for the regulation of core-clock proteins and metabolism. The circadian gene profile also showed variation based on age- and sex- differences, e.g., for participant 5 (male; age = 54 years), the majority of ~24 h genes depicted a peak expression after 16 h (Fig. 2a), while for participant 1 (male, age = 22 years), the majority of ~24 h genes depicted a peak expression at 12 h. WW Domain Containing Transcription Regulator 1 (*WWTR1*) gene, which is important for myoblast transformation in skeletal muscle was ~24 h rhythmic in both participants 1 and 5, however, its peak expression was at 22 h in participant 1 and at 4 h in participant 5. In case of participant 2 (female, age = 37 years), the majority of ~24 h genes depicted a peak expression after 12 h, while in case of participant 4 (male, age = 33 years), the majority of ~24 h genes peaked before 12 h (Fig. 2a). Nuclear Factor of Activated T Cells 5 (*NFAT5*) gene, which plays an important role in myoblast migration during skeletal myogenesis was ~24 h rhythmic in both participants 2 and 4, and its peak expression was at 19 h in the female participant and at 23 h in male participant. To further identify whether our genes of interest are affected by perturbations in clock elements in human skeletal muscle, we analysed RNA-seq dataset of control and *CLOCK* knockdown (*CLOCK*^KD^) fusioned myotube cells obtained from two healthy male donors (age = 60 and 62 years) and their clock-altered (using siRNA transfection) counterparts (Fig. 2b–d). Cullin 3 (*CUL3*) gene is an important component of the ubiquitin proteasome complex essential for proteolysis and is circadian expressed in primary myotube cells. We also identified differences in circadian properties and gene expression among the two donors. Cytochrome C Oxidase Subunit 6 C (*COX6C*) involved in mitochondrial respiratory chain showed phase shift in Donor 1 (>4 h; *q* < 0.05) while Transforming Growth Factor Alpha (*TGFA*) associated with cell proliferation showed phase shift (>4 h; *q* < 0.05) with *CLOCK*^KD^ in Donor 2 (Fig. 2b, c). A subsequent differential gene expression analysis showed upregulation in the core-clock gene *CRY1*, essential component of circadian machinery, and myogenesis related genes including Myogenin (*MYOG*), and Myosin Heavy Chain 3 (*MYH3*) that are essential for muscle differentiation, in at least one donor (Fig. 2d). While *CLOCK*^KD^ resulted in the downregulation of core-clock genes (*NR1D1, CLOCK, NR1D2*), and genes like Myocyte Enhancer Factor 2 A (*MEF2A*), which is crucial for skeletal muscle regeneration.

To assess circadian gene expression in WT mice and the impact of circadian clock alterations in mouse skeletal muscle, we analysed time-course microarray datasets derived from WT mice, muscle specific *Bmal1* knockout mice (*mBmal1*^KO^) and from a *Clock* mutant mice (*Clock*^mutant^) (Fig. 3). We observed significant alterations in circadian properties and gene expression profile among genes of interest resulting from the alterations of core-clock components (*Bmal1* or *Clock*) in mice skeletal muscle (Fig. 3).

In WT mice, we observed circadian expression of genes associated with skeletal muscle pathways such as *Atf4*, Fibroblast growth factor 2 (*Fgf2*), NDUFA4 mitochondrial complex associated like 2 (*Ndufa4l2*), and Fatty acid binding protein 3 (*Fabp3*). *Atf4* acts as a regulator of metabolic and redox processes while *Fgf2* is important for cytokine activity. *Ndufa4l2* is an integral part of mitochondrial respiratory chain and *Fabp3* participates in intracellular metabolism. The alteration in core-clock components (*Bmal1* or *Clock*) in mice resulted in a phase shift among genes of interest when compared to their control conditions (Fig. 3b, e, h). A total of 87 genes showed a phase shift (>4 h; *q* < 0.05) in either *mBmal1*^KO^ or in *Clock*^mutant^ vs. their controls (Fig. 3b, e, h). The core-clock genes *Nr1d2, Arntl* and metabolism associated gene, Protein kinase AMP-activated non-catalytic subunit beta 2 (*Prkab2*) showed a phase shift (>4 h; *q* < 0.05) in *Clock*^mutant^ vs. WT tissue (Fig. 3b). Moreover, we observed a phase shift in autophagy associated genes like GABA type A receptor associated protein like 1 (*Gabarapl1*) *in Clock*^mutant^ and Autophagy related 12 (*Atg12*) in *mBmal1*^KO^ vs. WT. We also observed upregulation of myonuclei associated gene Spectrin repeat containing nuclear envelope protein 1 (*Syne1*) in *Clock*^mutant^ vs. WT (Fig. 3c). *Myod1*, which is an important component of myogenesis was phase shifted and also differentially upregulated in *Soleus* muscle of *mBmal1*^KO^ vs. WT mice (Fig. 3e, f). Protein kinase C delta (*Prkcd*), associated with cell cycle progression showed phase shift (>4 h; *q* < 0.05) in both *Tibialis anterior* muscle of *mBmal1*^KO^ (Fig. 3h) and *Clock*^mutant^ mice datasets. *Cry2* significantly lost rhythmicity (*q* < 0.05) due to *mBmal1*^KO^, while *Myod1* and other clock-related genes (*Nr1d1*, *Per2*, D-box binding PAR BZIP transcription factor (*Dbp*)) showed significant rhythmicity loss in *Clock*^mutant^ vs. WT (*q* < 0.05).

Our analysis identified several genes, out of the predefined genes of interest, which depicted circadian expression in the datasets derived from the muscle biopsies of human participants and WT mice. In at least one dataset of the healthy participants, we observed circadian expression of muscle signalling-associated genes like *SUN2*, Heme Oxygenase 1 (*HMOX1*), and *WWTR1* and core-clock gene *RORA*. In WT mice datasets, we also observed circadian expression of *Sun2*, *Hmox1*, *Wwtr1*, and *Rora*. Altogether, our results showed that the genes of interest are clock regulated in both human and mouse skeletal muscle, and perturbations of core-clock elements affect their circadian properties.

### Extreme gravity environmental conditions induce molecular alterations in core-clock elements and skeletal muscle-associated pathways

Microgravity exposure can result in decreased muscle strength and endurance with a risk of muscle atrophy, which raises a major concern for astronauts on prolonged space missions^70^. To identify alterations in clock- and skeletal muscle- associated pathways due to spaceflight, we analysed omics datasets derived from mice sent to Space for 11 days (GSE10533), 30 days (GSE94381, GSE80223), 33 days (GSE156321), or 37 days (GLDS-99/-101/-103/-104/-105) and their respective ground controls. Current available datasets from spaceflight-based omics studies are derived from a single time point sampling (e.g., post spaceflight vs. ground controls), which limits circadian investigation. To partially overcome such limitation, we carried out differential expression analysis and evaluated alterations in our genes of interest in terms of average expression.

**Fig. 4:**
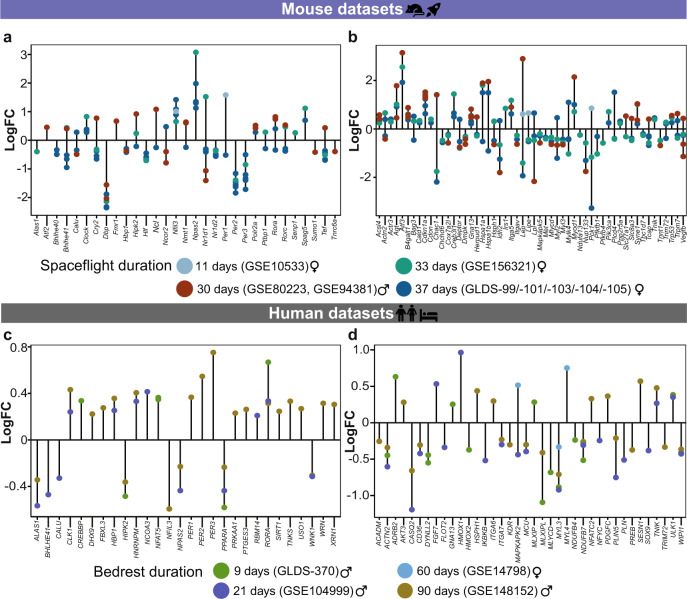
Strength of alterations in clock- and muscle- related genes correlates with the duration of spaceflight/bedrest in mammalian skeletal muscle. To explore the differences in differential gene expression based on the duration of spaceflight or bedrest, we grouped the datasets accordingly. The groups for spaceflight datasets were 11 days (GSE10533), 30 days (GSE80223, GSE94381), 33 days (GSE156321), and 37 days (/GLDS-99/-101/-103/-104/-105). The groups for bedrest datasets were 9 days (GLDS-370), 21 days (GSE104999), 60 days (GSE14798), and 90 days (GSE148152). Lollipop plots represent the distribution of differentially expressed clock-regulated genes (NCRGs) and muscle related genes due to spaceflight (**a**–**b**) and bedrest (**c**–**d**), respectively.

Bedrest, which is an analogue to spaceflight, also induces similar alterations leading to the impairment of muscle functional capacity^71^. We identified alterations in clock- and skeletal muscle-associated pathways in bedrest conditions, using microarray datasets derived from males subjected to bedrest for 9 days, 21 days, and 90 days and females subjected to bedrest for 60 days. We further categorized the spaceflight and bedrest datasets based on their duration (Fig. 4), and observed differential regulation among a pre-defined set of clock-related genes (NCRGs^26^) in at least one of the spaceflight datasets (Fig. 4a). A large number of skeletal muscle-associated genes were differentially expressed in the murine spaceflight datasets derived from 30 days (548 genes in at least one of the datasets), 33 days (430 genes), or 37 days (550 genes in at least one of the datasets) duration when compared to 11 days (61 genes) dataset. We selected for visualization genes that showed the same pattern (up-/down-regulation) in 30 days, 33 days, or 37 days spaceflight groups and then plotted the variation of those same genes in 11 days spaceflight dataset (Fig. 4b). The complete list of differentially expressed genes in each of the spaceflight datasets is provided in Supplementary Dataset 5.

For bedrest datasets, we analysed the differentially expressed NCRGs set and skeletal muscle specific genes in at least one of the datasets (Fig. 4c, d). Less number of genes were differentially expressed in females post 60 days bedrest (24 genes) compared to males subjected to 9 days (192 genes), 21 days (147 genes), or 90 days (250 genes) bedrest datasets. Core-clock genes like *Per1* and *Rora* were significantly upregulated and CCGs like 5’-Aminolevulinate Synthase 1 (*Alas1*), Homeodomain Interacting Protein Kinase 2 (*Hipk2*), Calumenin (*Calu*), Basic Helix-Loop-Helix Family Member E41 (*Bhlhe41*) were significantly downregulated in at least one of the spaceflight datasets (Fig. 4a). For bedrest datasets, the same set of genes (*PER1*, *RORA*, *ALAS1*, *HIPK2*, *CALU*, *BHLHE41*) were also downregulated (Fig. 4c). *Alas1* is associated with glucose intolerance in skeletal muscle and *Hipk2* is associated with cell proliferation and inflammation in skeletal muscle. *Calu* encodes for calcium ion binding, essential for skeletal muscle contraction, and *Bhlhe41* is known to inhibit inflammation and adipogenic differentiation in skeletal muscle. While, *Per2* was significantly downregulated in at least one of the spaceflights and bedrest datasets, *Per3* was significantly upregulated in the same datasets pointing to a compensation mechanism in the core-clock to maintain robustness (Fig. 4a, c). Actinin Alpha 2 (*ACTN2*), which is crucial for muscle contraction was significantly downregulated in bedrest datasets, while *Actn2* showed both upregulation/downregulation in its expression depending on the spaceflight duration and skeletal muscle type (Fig. 4b, d). Long-term duration of spaceflight resulted in severe alterations in gene expression profile. Core-clock genes like *Arntl*, *Clock*, *Cry1*, and Neuronal PAS Domain Protein 2 (*Npas2*) were significantly upregulated while *Cry2*, *Nr1d2*, *Per2*, and *Per3* were downregulated post 33 and 37 days in spaceflight mice vs. their ground controls. In addition, we observed upregulation of skeletal muscle-associated genes like *Myod1*, *Nfat5*, and Fos Proto-Oncogene, AP-1 Transcription Factor Subunit (*Fos*) and downregulation of *Dbp* post 30, 33, and 37 days in spaceflight (Fig. 4a, b). *Fos* gene is also known to be associated with aging phenotype in humans, as well as in mice^72^. Cyclin dependent kinase inhibitor 1 A (*Cdkn1a*) known to function as a regulator of cell cycle progression was significantly upregulated and Peroxisome proliferator activated receptor alpha (*Ppara*) associated with aging and metabolism was significantly downregulated in all spaceflight datasets, independent of duration (Fig. 4b).

We observed a higher number of differentially expressed genes in the datasets derived from long-term spaceflight duration (Fig. 4a, b). To investigate how many differentially expressed gene sets seen in long-term spaceflight datasets (37 days; GLDS-99/-101/-103/-104/-105) also exhibited circadian profile in WT mouse datasets, we carried out an intersection between the gene sets. Overall, 11207 genes showed ~24 h rhythmicity in at least one of the WT mouse circadian datasets (GSE54650, GSE3746, GSE43071) and 5017 genes were differentially expressed in at least one of the long-term spaceflight datasets. Out of 5017 genes, 3001 genes were circadian expressed in at least one of the WT mouse datasets. Within the predefined genes of interest, 1292 genes showed circadian expression in at least one of the WT mouse datasets. 760 genes of interest showed differential expression in at least one of the long-term spaceflight datasets and out of those, 550 genes were ~24 h rhythmic in at least one of the WT mouse circadian datasets. The core-clock elements *Arntl*, *Clock*, *Cry1/2*, *Npas2*, *Nr1d1/2*, *Per1/2/3*, *Rora/Rorc* and myogenesis associated genes like *Myod1*, *Myh3/9/10*, and *Atf4/5/6* were differentially expressed in spaceflight datasets and were ~24 h rhythmic in WT mouse circadian datasets. Furthermore, a Pearson correlation analysis showed variation between gene pairs depending on the skeletal muscle type (Supplementary Fig. 1a). We observed a significant negative correlation between *Cry1* and *Cry2* in ground controls (GLDS-99: correlation coefficient (r) = −0.823, *p* = 0.04; GLDS-103: r = −0.951, *p* = 0.003) while in the spaceflight groups *Cry1* and *Cry2* were positively correlated though non-significant (GLDS-99: r = 0.452; GLDS-103: r = 0.543, *p* > 0.05). Furthermore, *Clock* showed a negative correlation with *Cry2* gene in the ground controls (GLDS-104: r = −0.456; GLDS-105: r = −0.83, *p* = 0.04) while in the spaceflight group we observed a weaker negative correlation though non-significant between *Clock* and *Cry2* (GLDS-104: r = −0.042; GLDS-105: r = −0.456, *p* > 0.05; Supplementary Fig. 1a).

In addition, to investigate whether artificial hypergravity conditions can alter the molecular profile compared to spaceflight, we analysed proteomics datasets derived from mice subjected to hypergravity (3 g) for 28 days vs. their controls under normal gravity (1 g). The proteomics datasets related to spaceflight also showed a smaller number of differentially expressed proteins post 14 days (2 proteins) and 30 days (307 proteins in at least one dataset). Ryanodine receptor 3 (*Ryr3*) gene which encodes for an ion channel crucial for muscle contraction was significantly downregulated post 37 days in spaceflight, however, its protein was significantly upregulated in hypergravity datasets, but did not show significant differential expression in the spaceflight proteomics datasets. Taken together, our results showed that spaceflight alters the clock and skeletal muscle-associated pathways and that these alterations directly correlate with spaceflight duration. Moreover, bedrest being the analogue of spaceflight also showed similar discrepancies at the transcriptomic level.

### Age-induced circadian alterations in the expression profile of core-clock genes and skeletal muscle-associated pathways correlate with spaceflight induced gene expression alterations

Aging results in the gradual loss of muscle mass and its functional capacity, resulting in increased risk of sarcopenia^73^. In humans, comparisons between younger and older individuals reveal that fast-twitch fibres are smaller in the older, whereas slow-twitch fibres are less affected^74^. Aging brings a gradual decrease in size/volume of muscle fibres, and a replacement by fat and connective tissue^74^. Most importantly, aging is associated with impaired expression of core-clock genes^75^.

**Fig. 5:**
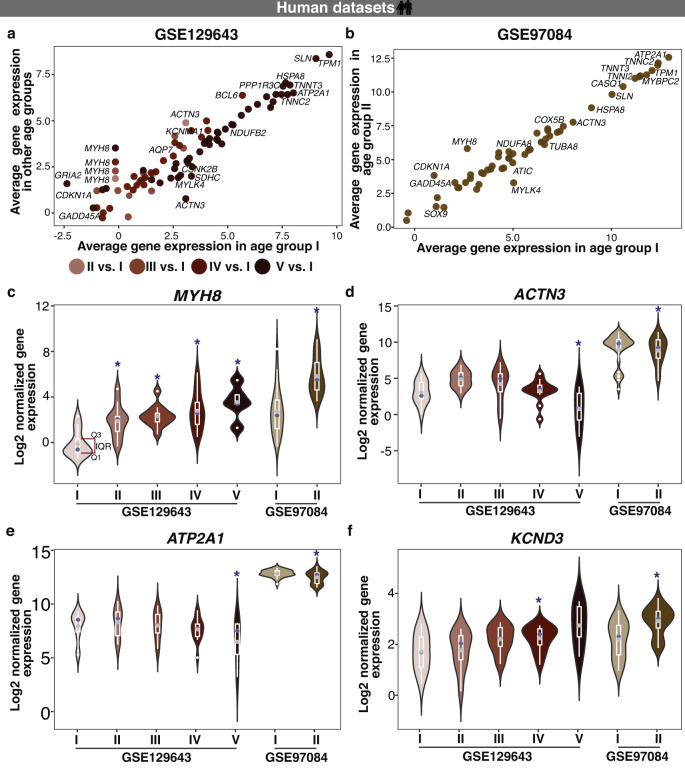
Aging on Earth results in differential expression of clock- and skeletal muscle-related genes in humans. Bubble plots represent (**a**) the average gene expression for differentially expressed genes in at least one age-group (Age group II = 35–49 years, III = 50–64 years, IV = 65–79 years, and V = 80 + years vs. Age group I = 20–34 years; GSE129643), that were also differentially expressed (**b**) in an independent age-related dataset (Age group II = 65–75 years vs. Age I = 20–30 years, GSE97084). Within the subset of differentially expressed genes of interest (**c**) *MYH8* exhibited age associated changes in the expression profile in all age groups vs. age group I (20–34 years; GSE129643) and in age group II (65–75 years) vs. I (20–30 years) in GSE97084 dataset, (**d**) *ACTN3* and (**e**) *ATP2A1* showed significant downregulation in the Age group V (80 + years; GSE129643) and in age group II (65–75 years; GSE97084) compared to their reference groups, and (**f**) *KCND3* showed significant upregulation in age group IV (65–79 years; GSE129643) and in age group II (65–75 years; GSE97084) vs. their reference groups (* represents the age groups that showed significant differential expression vs. reference group; Q1: 1st quartile; Q3: 3rd quartile; top and bottom whiskers: upper and lower adjacent values calculated as Q3 + 1.5IQR and Q1–1.5IQR, respectively; grey dots represent the mean value and blue dots represent the median value).

To investigate whether aging on Earth results in similar alterations among the genes of interest as seen during the spaceflight, we analysed RNA-seq datasets derived from muscle biopsies of one set of healthy subjects grouped according to age (GSE129643; Age group I = 20–34 years, Age group II = 35–49 years, Age group III = 50–64 years, Age group IV = 65–79 years, and Age group V = 80+ years), and an independent set of healthy subjects distributed in two age groups (GSE97084; Age group I = 20–30 years, Age group II = 65–75 years). Even though accumulating evidence points to alterations of circadian rhythms, and circadian regulated molecular and physiological processes with aging^75^, no publicly available time-course datasets from skeletal muscle samples in an aging scenario were found in the literature. We thus carried out differential expression analysis to investigate significant changes in average gene expression within clock and skeletal muscle-associated pathways. A total of 76 genes were differentially expressed in at least one of the age groups vs. age group I (Fig. 5a), and out of those, 58 genes were also differentially expressed in the other aging study dataset (Fig. 5b). We observed significant upregulation of Myosin Heavy Chain 8 (*MYH8*) in all aging groups in both datasets. *MYH8* is an actin-based motor protein involved in skeletal muscle contraction, cell migration and cell adhesion (Fig. 5c). This MyH type of isoform is expressed mainly during foetal development, but also during muscle regeneration. Its expression rapidly diminishes in adult cells. Dominant mutations in that gene lead to Trismus and pseudocamtodactyly syndrome, a congenital contracture of hands, feet and jaw, as well as hand and foot deformities^76,77^. Whereas, genes like *ACTN3* and *ATP2A1* were significantly downregulated in age group V vs I (GSE129643) and in age group II vs I (GSE97084) datasets (Fig. 5d, e). *ACTN3* encodes for alpha-actinin-3, which plays a major role in skeletal muscle. It is expressed predominantly in fast-twitch, glycolytic muscle fibres and acts as a binding protein with a structural role at the sarcomeric Z-line. Thus, it influences the force production and force transmission capabilities of muscle fibres. Moreover, *ACTN3* regulates muscle metabolism. *ATP2A1* encodes an enzyme sarcoplasmic/endoplasmic reticulum (SR) calcium ATPase 1 (SERCA1), which is responsible for the translocation of calcium ions from the cytosol into the SR of skeletal muscle. The SR serves as a store for calcium in muscle and can initiate muscle contraction by releasing calcium through the ryanodine receptor type-1 (RyR1), as well as muscle relaxation by active reuptake of calcium by SERCA. SERCA isoforms are distributed differently in skeletal muscle. SERCA1a can be found mainly in fast twitch muscle of adults whereas SERCA1b is predominantly expressed in foetal skeletal muscle. SERCA2 is predominantly expressed in slow twitch muscles (e.g. *Soleus*). Mutations in *SERCA* cause Brody’s disease that manifests in severe muscle cramps and exercise induced impairment of skeletal muscle function^78^. *KCND3*, which encodes the voltage-gated potassium ion channel was significantly upregulated in age group IV vs. I and also in age groups II vs. I (GSE97084 dataset) (Fig. 5f).

In addition, *CDKN1A*, SRY-Box Transcription Factor 9 (*SOX9*), Potassium Calcium-Activated Channel Subfamily M Alpha 1 (*KCNMA1*), were upregulated in age group IV vs. I (in GSE129643 dataset) and in age group II vs. I (in GSE97084 dataset). *SOX9* is important for musculoskeletal development^79^ while *KCNMA1* encodes for a voltage and calcium dependent potassium channel important for myoblast differentiation, and contributes to the cardiac transient outward potassium current during the repolarizing phase in the cardiac muscle cell contraction cycle^80^. Myosin Light Chain Kinase Family Member 4 (*MYLK4*), important for muscle strength and stiffness was significantly downregulated in age groups V vs. I (GSE129643 dataset) and also in age groups II vs. I (GSE97084 dataset).

Higher number of differentially expressed genes of interest were seen in comparisons between extreme age groups (GSE129643: Group V vs. Group I; GSE97084: Group II vs. Group I). Overall, 5263 genes showed ~24 h rhythmicity in at least one of the healthy human circadian datasets (GSE108539, GSE109825) and 3368 genes were differentially expressed in at least one of the extreme age group datasets. Out of 3368 genes, 1037 genes were circadian expressed in at least one of the human control datasets. Within our set of pre-defined genes of interest, 688 genes showed circadian expression in at least one of the healthy human datasets. 455 genes of interest showed differential expression in at least one of the aging datasets and out of those, 165 genes were ~24 h rhythmic in at least one of the healthy human circadian datasets. Clock elements such as *ARNTL*, *BHLHE40* and genes associated to skeletal muscle pathways like *FGF2/7*, *MYH3* were differentially expressed in extreme age groups datasets and were ~24 h rhythmic in human control datasets. Furthermore, we carried out a Pearson correlation analysis to evaluate the differences in the core-clock network in aging condition datasets (GSE129643: Group V vs. Group I; GSE97084: Group II vs. Group I; Supplementary Fig. 1b, c). In GSE129643 dataset, *ARNTL* showed negative correlation with *PER1* in Group I (age = 20–34 years; r = −0.357, *p* = 0.006) whereas in Group V (age = 80+ years), *ARNTL* showed weaker though non-significant negative correlation with *PER1* (r = -0.43, *p* > 0.05; Supplementary Fig. 1b). In GSE97084 dataset, we observed positive correlation between *NR1D2* and *RORA* in Group I (age = 20–30 years; r = 0.867, *p* < 0.0001). While, *NR1D2* and *RORA* showed negative (non-significant) correlation in Group II (age = 65–75 years; r = −0.155, *p* > 0.05) in GSE97084 dataset (Supplementary Fig. 1c).

To identify possible common molecular dysregulation between aging and spaceflight datasets, we compared the differentially expressed genes in both scenarios. A total of 129 genes were significantly upregulated in at least one of the aging condition datasets and in at least one of the spaceflight datasets. These included Calsequestrin 2 (*CASQ2*), and Fibroblast Growth Factor 1 (*FGF1*). *CASQ2* acts as a calcium store for muscle function and *FGF1* is involved in muscle development and regeneration. A total of 113 genes were significantly downregulated in at least one of the aging condition datasets and in at least one of the spaceflight datasets. Among the downregulated genes were Nitric Oxide Synthase 1 (*NOS1*), *FABP3*, and *FGF13*. *NOS1* generates nitric oxide (NO) which is known to function as a gaseous and highly diffusible neurotransmitter in the brain but also as a co-mediator for skeletal muscle contractility or vascular smooth muscle wall dilation, *FABP3* is associated with the maintenance of muscle strength, and *FGF13* is important for skeletal muscle differentiation. Taken together, these results show molecular alterations in clock- and skeletal muscle-associated pathways, in human skeletal muscle, in the context of aging, which resemble gene expression alteration observed in spaceflight in murine datasets.

### Extrinsic Zeitgebers like fasting and physical activity alter circadian regulation of skeletal muscle related elements

Physical activity or exercise are reported as effective countermeasures against general effects of aging (e.g., sarcopenia) and also play a major role in the prevention of musculoskeletal disorders^81^. Fasting improves metabolic health and is associated with the loss of lean body mass^82^. Yet, it is unclear whether exercise based on time or intensity, or fasting can help reverse the molecular alterations observed due to spaceflight, or aging on Earth. To investigate this hypothesis, we analyzed published RNA-seq datasets derived from mice subjected to basal (45% of aerobic capacity), moderate (55% of aerobic capacity), or high intensity (100% of aerobic capacity), running exercises at ZT (Zeitgeber Time) 14 (2 h within dark phase, termed as early) or ZT22 (10 h within dark phase, termed as late) (GSE117161), rats with low and high capacity running (GSE155230), mice subjected to running for predefined durations vs. sedentary groups (GSE178262), and human datasets derived from two age groups (Young: 20–30 years and Older: 65–75 years) subjected to 12 weeks of resistance (strength exercise using free weights) or high intensity (aerobic exercise using treadmill) or combined (resistance plus high intensity) exercise vs. pre-exercise (baseline; GSE97084). Based on our selection criteria, we retrieved one published time-course dataset based on fasting and mammalian skeletal muscle (GSE107787), where the skeletal muscle samples were obtained from mice subjected to 24 h fasting vs. *ad libitum* group.

**Fig. 6:**
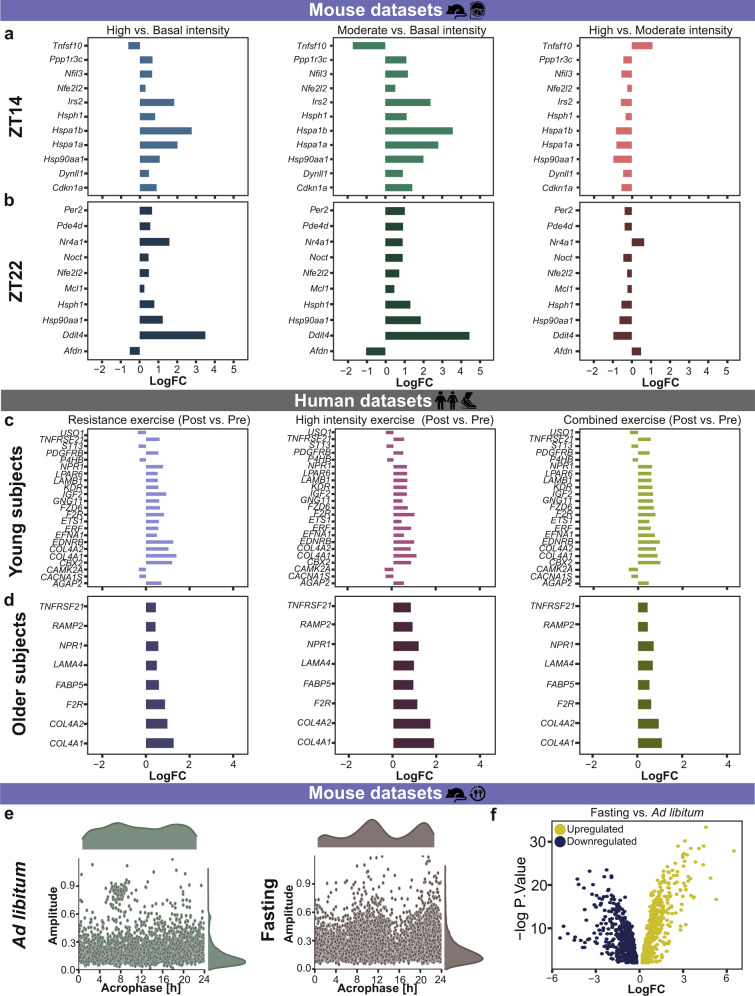
Extrinsic Zeitgebers like exercise timing and intensity, and fasting affect differential expression of clock- and muscle-related genes in mammalian skeletal muscle tissue. Histogram plot depicts the common differentially expressed genes independent of exercise intensity (**a**) at ZT14 and (**b**) at ZT22 in mouse skeletal muscle, (**c**) in young subjects and (**d**) in older subjects. **e** Scatter plot represents the circadian properties (amplitude, acrophase) of ~24 h genes after *ad libitum* and fasting. **f** Volcano plot represents significantly up- or down-regulated genes of interest between *ad* *libitum* and fasting groups.

The differentially expressed genes in mice skeletal muscle varied based on the type and time of exercise (Supplementary Fig. 2). To examine the molecular changes with type and time of exercise, we extracted the common differentially expressed genes between high vs. basal, moderate vs. basal, and high vs. moderate intensity exercise at ZT14 (Fig. 6a) and at ZT22 (Fig. 6b). A total of 11 common genes were differentially expressed for all exercise intensities at ZT14 (Fig. 6a), out of these, were NFE2 like BZIP transcription factor 2 (*Nfe2l2*), important for mitochondrial biogenesis induced by exercise in skeletal muscle, and *Cdkn1a*. A total of 10 common genes were differentially expressed for all exercise intensities at ZT22 (Fig. 6b), out of these were *Nfe2l2* and *Per2*. *Nfe2l2* was significantly upregulated in high vs. basal and moderate vs. basal intensity exercises and at both ZT14 and ZT22. Furthermore, we also investigated the individual impact of different exercise intensities at ZT14 and ZT22. Myonuclei associated genes *Dctn1* and *Dctn3* were significantly downregulated in moderate vs. basal intensity exercise at ZT14. While, *Dctn1* was significantly upregulated in high vs. moderate intensity exercise at ZT22. In high vs. basal intensity groups, *Dctn4* was upregulated at ZT14 while *Cry1* and *Per2* were upregulated at ZT22. In moderate vs. basal intensity groups, *Myh11* was significantly upregulated. When we analysed a different time dependent exercise dataset (GSE178262), *Dctn1* and *Myh11* were also upregulated in a group subjected to exercise at ZT12 vs. ZT0. In addition, *Myh11* was also significantly upregulated in the dataset derived from rats with high running capacity vs. rats with low running capacity, representing the impact of exercise type on skeletal muscle.

The differentially expressed genes found in human skeletal muscle varied based on the type of exercise and age of the subjects (Supplementary Fig. 3). To examine the impact of exercise type on clock- and skeletal muscle-associated pathways, in an age-dependent manner, we analysed the data from young and older human subjects who underwent three types of exercise (resistance, high intensity, and combined) vs. their profile before exercise. A total of 23 common genes were differentially expressed in the young group subjected to different types of exercise (Fig. 6c) and out of these, were genes like ETS2 Repressor Factor (*ERF*) and Insulin like growth factor 2 (*IGF2*). *ERF* is important for cellular proliferation and *IGF2* is critical for normal muscle growth, and is also associated with aging hallmarks^83^ (Fig. 6c). A total of 8 genes were differentially expressed in the older group subjected to different exercise type (Fig. 6d), including Laminin Subunit Alpha 4 (*LAMA4*) and *FABP5*. We also observed differences in differentially expressed genes based on the age of the subjects. For example, *MYH9* and Transforming Growth Factor Beta 1 (*TGFB1*) were upregulated in young subjects post resistance exercise while were not differentially expressed among older subjects. The voltage ion channel gene *VDAC1* was significantly downregulated among young subjects post high intensity exercise. The core-clock genes *PER2*, *PER3*, *CRY2*, and *NR1D1* were significantly downregulated among older subjects in at least one of the exercise groups vs. pre-exercise. In addition, we also extracted the set of genes that were differentially expressed among young, as well as in older subjects depending on the exercise type. A total of 33 common genes were differentially expressed in both age groups during resistance exercise, out of which, there were skeletal muscle-associated genes like Emerin (*EMD*) and Leimodin 1 (*LMOD1*). *EMD* is important for cytoskeleton organization and its absence or defect is seen among Emery-Dreifuss muscular dystrophy^84^, while *LMOD1* is associated with muscle contraction. A total of 80 genes were differentially expressed in both the young and older subjects during high intensity exercise, out of which were *ATF1*, linked with hypoxia response in muscle, and *MYH10*, crucial for myogenesis. 22 genes were common between young and older subjects from combined exercise, including myonuclei related gene *DCTN1* and mitochondrial gene *MT-CO2*.

Furthermore, we investigated the alterations in the molecular profile of clock- and skeletal muscle- associated pathways in mice due to fasting (Fig. 6e, f). Interestingly, we observed higher number of ~24 h rhythmic gene sets in the fasting group (723 genes) vs. *ad libitum* group (337 genes). Moreover, we also observed an overall difference in the acrophase and amplitude between the two groups (Fig. 6e). *Myh3*, *Myh11*, *Dctn2*, and *Dctn4* were ~24 h rhythmic in the fasting group when compared to the *ad libitum* group. A total of 1131 genes showed differential expression in fasting vs. *ad libitum* group, and out of them, 363 genes were downregulated, e.g., *Myod1, Cry1, Per3*, and *Dbp* and 768 genes were upregulated, e.g., *Per1, Myh3, Dctn2, Npas2, Rorc, Mtor, Sun1*, and *Cry2* (Fig. 6f).

**Fig. 7:**
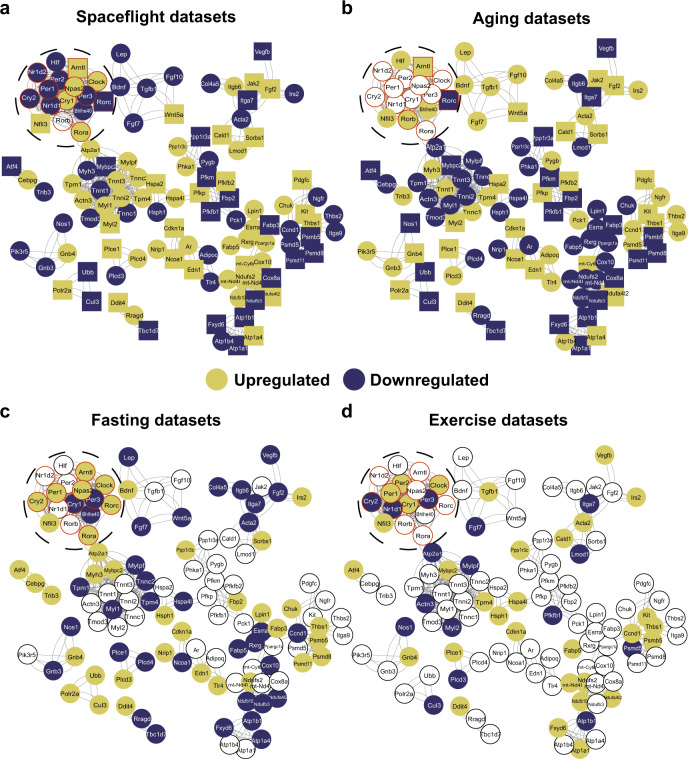
Network representation of differential expressed genes altered in both spaceflight and aging datasets and their association with core-clock elements. 206 common genes were differentially expressed in at least one of (**a**) the long-term spaceflight datasets (37 days) and (**b**) in extreme age groups (older vs. young). In addition, core-clock elements (bold circles with red border) were also added to the network to explore the regulatory role of circadian clock machinery. Network analysis was carried out using STRING and MCODE, cytoscape based plugins. The same gene sets were plotted for (**c**) fasting and (**d**) exercise datasets. The gene sets were color coded based on their up- or down-regulation compared to their respective controls. Set of genes that depicted similar pattern (up or down-regulation) in spaceflight and aging datasets were shown as rectangles. Genes with no differential expression are represented as white circles.

We further identified specific molecular alterations by retrieving the gene sets that were differentially expressed in at least one of the long-term spaceflight datasets (37 days) and in the older age group (Age group V vs. I (GSE129643) or in Age group II vs. I (GSE97084)). A total of 206 common genes were differentially expressed between long-term spaceflight and aging condition datasets. Using Cytoscape plugins (STRING and MCODE), we visualized the interaction between the obtained genes in spaceflight, aging, exercise, and fasting condition datasets, and included core-clock genes to the network even if they were not differentially expressed in the two conditions (Fig. 7a–d). Circadian clock related genes including *Arntl, Bhlhe40, Cry2, Rorc* and skeletal muscle-associated genes like Wnt family member 5A (*Wnt5a*), *Atf4*, Vascular endothelial growth factor beta (*Vegfb*), *Cdkn1a*, *Myl2*, *Ndufa4l2*, *Fabp3*, *Ndufb3, Fgf2, Cul3, Acta2*, Leptin (*Lep*)*,* *Actn3, and Heat shock protein family A member 8* (*Hspa8*) were differentially expressed in at least one of the long-term spaceflights, and aging condition datasets. *Arntl* was upregulated in the aging, spaceflight, and fasting condition datasets. In contrast, *Rorc* was downregulated in spaceflight (post 37 days) and aging condition datasets, while upregulated in the fasting condition dataset (Fig. 7). *Myl2* was significantly upregulated in spaceflight and aging condition datasets, but upregulated in at least one of the exercise condition datasets. *Wnt5a*, which is known to promote myogenesis was upregulated in Space and aging condition datasets while, its expression was downregulated in fasting condition datasets. *Atf4/ATF4* known to be associated with muscle atrophy and also with aging phenotype^85^ was significantly downregulated in Space and aging-related datasets, while its expression was upregulated in fasting condition dataset and significantly downregulated in young human participants subjected to 12-weeks of high-intensity exercise. Altogether, our analysis identified a set of genes with molecular profiles altered during spaceflight (especially long-term) or aging condition datasets, which can potentially be regulated by using external Zeitgebers to recover altered phenotypes associated with circadian dysregulation.

## Discussion

The European Space Agency (ESA) and the National Aeronautics and Space Administration (NASA) recently started to use Space omics technology in cells, animals and astronauts in spaceflight^86–88^. A number of clock genes with epigenetic modifications was reported through the fundamental Space omics research carried out by the NASA GeneLab project^87^. Such microgravity-triggered epigenetic modifications associated with clock genes was also seen in late pregnant rats^89^, thereby supporting the notion of epigenetic modifications triggered by microgravity in cells, small animals, and during development^89^. In a recent study using transcriptomics data, asynchrony among clock genes were reported in different peripheral tissues of space flown rodents^52^. In the retina from 37 days space flown mice onboard the ISS, for example, a number of genes were differently methylated with spaceflight vs. ground control (sex and age-matched) suggesting that microgravity decelerated the retinal clock at both epigenomic and transcriptomic levels with potential impact on eye-related disorders such as spaceflight-associated neuro-ocular syndrome including optic disc edema, seen among astronauts^90^. However, detailed molecular insights regarding circadian dysregulation specific to skeletal muscle due to different gravity environments is still lacking and is most relevant.

Circadian rhythms play a regulatory role in the skeletal muscle via processes like myogenesis, transcription of specific genes, and metabolism^5^. Our analysis strengthens such results and shows circadian expression of several genes that are important for skeletal muscle mass, as well as in elements of the circadian clock network in mammalian skeletal muscle datasets. Myonuclear positioning is important for normal muscle function and its dysregulation has been linked to pathologies like muscle dystrophies^91^. *SUN2* gene is crucial for myonuclear positioning and its dysregulation affects cytoskeleton organization^92^. We observed clock-regulated expression of *SUN2* gene in both human and WT mouse datasets. However, *SUN2* circadian expression was not seen in clock manipulated mammalian datasets, highlighting a role for the clock in myonuclear organization. *MYOG* gene is essential for skeletal muscle homeostasis, and it also showed circadian expression in healthy human circadian datasets. *Myog* has been reported to regulate myocyte fusion, and its loss led to alterations in mTOR signalling in muscle cells^93^. Moreover, clock-regulated expression of *MYOG* was reported previously in rabbits, and night-restricted feeding also resulted in its increased expression^94^. Skeletal muscle differentiation is essential for development where *MYH3* plays an important role^95^. Interestingly, our results show that *MYH3* is circadian in skeletal muscle in both human and WT mice datasets. Such examples of clock-regulated genes strengthen the role of the circadian clock in skeletal muscle development.

In this study, we found that several genes undergo similar alterations due to spaceflight, as well as a result of aging on Earth. Extrinsic factors like exercise or fasting represent possible interventions that can help to minimize the molecular alterations resulting from spaceflight and/or aging on Earth. To overcome the lack of time-course datasets for spaceflight and aging conditions, we carried out differential expression analysis between space flown/aged groups and their respective controls, and used the circadian datasets derived from human and mouse skeletal muscle to visualize possible circadian variations in gene expression. Our analysis showed that the skeletal muscle-associated pathways are clock regulated in both human and mouse datasets, and core-clock alterations can result in similar molecular pattern as seen in spaceflight datasets. For example, skeletal muscle-associated genes like *Nfat5*, *Fos*, and *Myod1* showed circadian expression in WT mouse and healthy human datasets while their rhythmicity was changed in clock altered tissues, in a mouse model. *Nfat5* showed loss of rhythmicity in both the mouse core-clock altered datasets, and its expression was upregulated in at least one of the spaceflight datasets. *Nfat5* is important for myoblast migration required for skeletal muscle regeneration and its deletion in a mouse model depicted a defect on muscle regeneration^96^. *Myod1* showed phase shift with *mBmal1*^KO^ vs. WT and its expression was also upregulated in both *mBmal1*^KO^ and in at least one of the spaceflight datasets. *Myod1* is the key regulator of skeletal muscle differentiation^97^ and its deletion in mouse skeletal muscle stem cells resulted in the inhibition of skeletal muscle regeneration^98^. We observed similar alterations in clock genes, such as *Cry2, Nr1d2, Dbp, and Per2*, which were downregulated due to both the mutation in *Clock* gene and also due to spaceflight. *Dbp* downregulation due to spaceflight was reported previously in different peripheral tissues from spaceflight experiments^51,52^. Interestingly, in datasets derived from patients with neurodegenerative diseases like Parkinson’s, an upregulation of *DBP* was reported among patients as compared to the healthy control groups^99^. This highlight potential implications of spaceflights in neurodegeneration and further research in this direction will be needed. Our observations point towards common molecular alterations resulting due to spaceflight and molecular perturbations of the circadian system and putative health impact for astronauts. Further studies are needed to pinpoint the specific impact of core-clock elements alterations on skeletal muscle signalling in mammalian tissue on Earth and in Space.

In addition, long-term spaceflights resulted in severe molecular alterations in clock- and skeletal muscle-associated pathways. In particular, the differential gene regulation observed during long-term spaceflight datasets resembled expression changes due to aging condition on Earth. In particular, skeletal muscle-associated genes like *Atf4*, *Ndufa4l2*, *Fabp3*, *Cul3*, *Fgf2*, and *Wnt5a* showed similar pattern (up-/down-regulation) in both spaceflight (post 37 days) and in aging condition datasets, as compared to the respective controls. Our analysis also shows a differential regulation of these genes found in fasting or exercise condition datasets, that can potentially serve as an external factor to minimize the circadian alterations in skeletal muscle. Moreover, several genes gained rhythmicity under fasting conditions, reflecting a tissue-specific and time-of-day dependent response to eating behaviour. Time-restricted feeding or caloric restriction may serve as a practicable alternative to minimize circadian alterations applicable to the daily routine of astronauts in Space. Indeed, the positive impact of time-restricted feeding on muscle function was reported both on *Drosophila*^100^ and rodents^101–103^ and may be a feasible intervention during spaceflights, also applicable to aging on Earth. Although fruit fly muscle pathology may not be comparable 1:1 to diseased vertebrate or mammalian skeletal muscle pathology, some of the obesity-induced changes reported in *Drosophila* muscle may serve as proof-of-concept for circadian rhythm-associated muscle dysfunction linked to extreme feeding conditions^100^. In fact, in 13 days space flown *Drosophila* (Shenzhou-9 spaceship) disrupted circadian rhythms resulted in altered lipid metabolism and life span^104^. In addition, caloric restriction, which involves time restricted feeding of less than 6 h has been shown to improve longevity in mammals, but so far not in *Drosophila*^105,106^. Applying such caloric restriction to astronauts may be less feasible due to their mission related energy balance, but it might still be an interesting approach to minimize age-related circadian dysregulation on Earth.

**Fig. 8:**
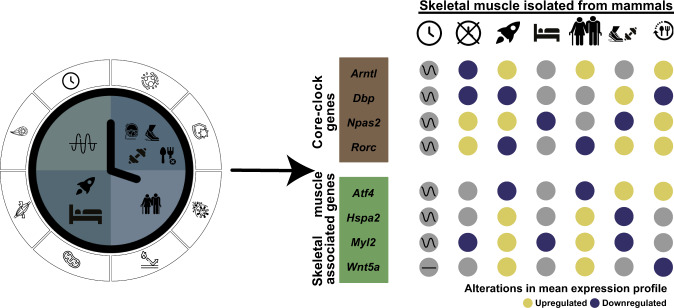
Graphical representation of the study and main results. This study mainly focused on investigating molecular changes in skeletal muscle-associated and circadian rhythm-associated pathways such as metabolism, myonuclei, myogenesis (left side; symbols) in healthy skeletal muscle, and altered conditions (i.e., clock manipulation, spaceflight, bed rest, aging, exercise, fasting) from left to right order. Core-clock genes and skeletal muscle-associated genes depicted similar patterns (upregulation: yellow; downregulation: blue) in gene expression profiles due to spaceflight and aging on Earth (e.g. *Rorc*, *Atf4*; right side). While exercise or fasting conditions showed opposite gene expression patterns in the same genes.

Previous studies, in healthy participants and athletes, reported the influence of exercise on skeletal muscle and performance based on the time of day^107–110^. Specific genes have been associated to performance of the skeletal muscle and thus are also interesting targets, which might be altered through exercise. Among those, *Atf4* was downregulated in Space and aging condition datasets while its expression was upregulated in fasting and exercise (based on the type) condition datasets. *Atf4* encodes a DNA-binding protein that functions as a leucine zipper transcription factor and plays an important role in mediating cellular stress responses. In immobilized mice, *Atf4* is highly expressed in skeletal muscle and induces muscle fibre atrophy^111^. Whereas, *Ndufa4l2* was upregulated in Space, aging and exercise condition datasets, its expression was downregulated under fasting conditions. *Ndufa4l2* encodes a protein that is a subunit of NADH dehydrogenase (Complex I) and part of the respiratory chain in mitochondria. Its expression can be induced by hypoxic stimuli and results in decreased mitochondrial respiratory activity in order to keep levels of reactive oxygen species (ROS) as low as possible, but also leads to less important intramuscular metabolites (e.g. adenine nucleotides, NAD + )^112^. *Fabp3* gene, associated with fatty acid metabolism, was downregulated in Space and aging condition while it was upregulated in fasting condition datasets. *FABP3* modulates age-dependent lipid remodelling and its overexpression can cause ER stress and defective protein synthesis in skeletal muscle^113^. *Fgf2*, which encodes the growth factor and signalling protein was significantly upregulated in spaceflight and aging condition datasets, while it was downregulated in fasting condition dataset. *Fgf2* plays an important role in the bone-muscle crosstalk and is mainly expressed by mesenchymal-derived stromal cells (e.g., fibroblasts/fibrocytes), osteoblasts but also skeletal myocytes. It can act as both promotor and inhibitor of osteoblastogenesis. In case of muscle injury, FGF-2, stored in the sarcoplasm of skeletal myofibers, is released and triggers an anabolic effect^114^. *Wnt5a* gene was downregulated in spaceflight and aging condition datasets while its expression was upregulated in fasting condition datasets. The Wnt signalling pathway is involved in cellular differentiation and proliferation in adult tissue. Wnt5a regulates the expression of *MyoD* and *Myf5* that play an important role in myogenesis of skeletal muscle^115^. Through our preliminary analysis, we highlight the potential applications of either exercise or fasting as a countermeasure against molecular alterations due to aging on Earth or long-term spaceflight. For example, in the case of *Ndufa4l2*, fasting can serve as a countermeasure to minimize the alterations in gene expression profile (Fig. 8). Nevertheless, due to the limited availability of circadian datasets from the literature a comprehensive evaluation of the molecular circadian profile in datasets from space flown mice and aged human vs. their controls remain to be established by further studies.

In future deep Space exploration, the duration and distance of Space travel impose great risks to the Crew´s health with the need to find smart countermeasures, thus mitigating such mission-related risks for both astronaut and work schedule. The outcome of our study supports the hypothesis of microgravity-triggered circadian clock changes in skeletal muscle-related pathways, found in studies with mice, bedrest or astronauts in spaceflight. Future experiments will be needed to further validate our findings and explore more insights to circadian regulation resulting from spaceflight using time-course experiments. Moreover, the current observations are retrieved from mouse and human studies and with different muscle types (slow vs. fast twitch), that are affected differently in microgravity conditions, and would be relevant to consider in following studies.

The obvious question will be how such knowledge may be translated to optimize countermeasures to mitigate or even prevent circadian dysregulation in future space missions. Monitoring the circadian rhythms will likely become an integral part of space missions, and new easy-to-use and non-invasive methods will be required for it^107^. Circadian rhythm-dependent muscle signalling and function nevertheless imposes a yet underestimated, but significant challenge not only for clinical cohorts suffering from various neuro-musculoskeletal disorders on Earth, but also for Space travellers health and performance in future missions with crewed spacecrafts and planetary habitats utilized for Deep Space and Planetary Exploration of Space Agencies to Moon and Mars.

## Methods

### Acquisition of publicly available RNA-seq datasets

Using a criterion of “skeletal muscle”, “mammalian tissue”, and “RNA-seq” on open source repositories (NCBI-GEO/ArrayExpress/NASA GeneLab), we obtained a total of 534 samples from 14 different datasets. Out of 14 RNA-seq datasets, 4 datasets were retrieved from healthy humans and 10 datasets were retrieved from mice.

Based on the human data, we collected RNA-seq datasets derived from:

1. *Vastus lateralis* (thigh) muscle biopsies collected from healthy subjects at 12:00 h–08:00 h, every 4 h (*n* = 9 (8 males and 1 female); Age = 29.9 ± 9.8 years; Accession number: GSE108539^116^);
2. *Gluteus maximus* (hip) muscle biopsies collected from healthy male subjects (*n* = 2; Age = 61.1 ± 1 years). Primary skeletal myoblasts were purified from the biopsies and differentiated into myotubes, which were transfected either with siControl or *siCLOCK*. siRNA transfection in cultured primary human skeletal myotubes resulted in the disruption of *CLOCK* rhythms, and also its downregulation compared to the untreated control cells^116^. Post-synchronization, samples were collected every 2 h from 0 h–48 h (Accession number: GSE109825^116^);
3. *Vastus lateralis* (thigh) muscle biopsies collected from healthy young and older subjects (sex and age matched) who underwent different exercise regimes. All subjects were randomly divided into: High-intensity interval exercise (HIIT; young (*n* = 14; Mean ± SD: 25.4 ± 4.3 years); older (*n* = 9; Mean ± SD: 70.7 ± 4.6 years)), Resistance exercise (RT; young (n = 11; Mean ± SD: 23.7 ± 3.5 years); older (n = 9; Mean ± SD: 70.3 ± 3.9 years)), and Combined exercise (CT: Resistance + high-intensity; young (*n* = 9; Mean ± SD: 26.3 ± 2.7 years); older (*n* = 8; Mean ± SD: 68.6 ± 3.4 years)). Biopsies were collected pre-exercise (baseline) and post-12-weeks exercise. *Vastus lateralis* (thigh) muscle biopsies were collected from young and older participants in the morning at 10 h (Accession number: GSE97084^117^);
4. *Vastus lateralis* (thigh) muscle biopsies collected from healthy male and female subjects (n = 58; Accession number: GSE129643^118^) from different age groups (20–34 years, *n* = 13; 35–49 years, *n* = 11; 50–64 years, *n* = 12; 65–79 years, *n* = 12; 80+ years, *n* = 10).

Based on the mouse model system, there were RNA-seq datasets derived from:

1. *Gastrocnemius* (calf) muscle collected from male C57BL/6 mice either subjected to *ad libitum* normal chow feeding (FED) or 24 h fasting (FAST) under 12 h light/12 h dark cycle. Samples were collected every 4 h from ZT0-ZT20 (*n* = 3 per time point per group; Accession number: GSE107787^119^). This is the only time course dataset available on feeding/fasting, based on our selection criteria;
2. *Gastrocnemius* (calf) muscle collected from low response exercise and high response exercise male rats before and after 8-weeks exercise program (Accession number: GSE155230^120^);
3. *Gastrocnemius* (calf) muscle from male C57BL/6 mice collected at ZT14 (early) and ZT22 (late) from basal, moderate, and high intensity running protocol (Accession number: GSE117161^121^);
4. *Gastrocnemius* (calf) and glycolytic quadriceps muscles collected from male C57BL/6Jrj mice with/without acute treadmill and chronic wheel running exercise at different times of the day (Accession number: GSE178262^122^);
5. *Gastrocnemius* (calf) muscle collected from female C57BL/6 N mice subjected to 33 days in spaceflight and their ground control (Accession number: GSE156321^123^). In addition, muscle samples were collected from un-injected mice post 33 days in spaceflight and their ground controls. The mice remained on-board of the ISS until the departure of SpaceX on January 7, 2020 and were delivered live to Explora Biolabs ∼32 h after splashdown in the Pacific Ocean (live animal return, LAR). The un-injected space flown and ground control datasets used in our analysis were retrieved from mice that were euthanized for analysis within the first 8 h after LAR^123^.
6. Different muscle tissues were collected from female C57BL/6 J mice subjected to 37 days in spaceflight (microgravity) and their ground controls, (namely 6) *Extensor digitorum longus* (foot lever) muscle (Accession number: GLDS-99),
7. *Gastrocnemius* (calf) muscle (Accession number: GLDS-101),
8. *Quadriceps* muscle (Accession number: GLDS-103),
9. *Soleus* (deep calf) muscle (Accession number: GLDS-104), and
10. *Tibialis anterior* muscle (Accession number: GLDS-105). For the mice used in the studies (6–10), carcasses were frozen in a pre-chilled Mini Cold Bag (MCB) before transfer to the MELFI freezer (−80 °C) and brought back to Earth where dissection was held. The mice in the ground control group also followed the same protocol. Total dissection time for each carcass was no longer than 30 min since collection of the first tissue.

The summary of omics datasets used in this study is provided in the Supplementary file 1.

### Acquisition of publicly available Microarray datasets

Using a criterion of “skeletal muscle”, “mammalian tissue”, and “Microarray” in open source repositories (NCBI-GEO/ArrayExpress/NASA GeneLab), we obtained a total of 485 samples from 10 different datasets. Out of 10 microarray datasets, four datasets were retrieved from healthy humans and six datasets were retrieved from mice.

Based on the human samples, we collected microarray datasets derived from:

1. *Soleus* and *Vastus lateralis* muscle biopsies collected from healthy female pre- and post-60 days bedrest (BR; Accession number: GSE14798^124^). The subjects were randomly divided into: BRC (Age (Mean ± SD) = 34 ± 3 years; *n* = 8; bedrest alone), BRE (Age = 32 ± 3 years, *n* = 8; bedrest combined with exercise), and BRN (Age = 29 ± 3 years, *n* = 8; bedrest combined with protein supplements);
2. *Vastus lateralis* muscle biopsies collected from healthy male subjects pre- and post-21 days normoxic bedrest (Age (Mean ± SD) = 27 ± 6 years, *n* = 14; Accession number: GSE104999^125^);
3. *Vastus lateralis* muscle biopsies collected from healthy male pre- and post-90 days bedrest (Accession number: GSE148152^126^). The subjects were randomly divided into: BR (Age (Mean ± SD) = 33 ± 5 years; *n* = 9; bedrest alone) and BRE (Age (Mean ± SD) = 32 ± 4 years; *n* = 12; bedrest combined with exercise);
4. *Vastus lateralis* muscle biopsies collected from healthy male pre- and post-9 days bedrest (Age = 24–27 years, Accession number: GLDS-370). In addition, the subjects completed 4-weeks retraining program post-BR and biopsies were collected.

Based on the mouse samples, there were microarray datasets derived from:

1. *Gastrocnemius* (calf) muscle collected from male C57BL/6 mice every 2 h from CT18-CT64 (Accession number: GSE54650^9^);
2. *Tibialis anterior* and *Soleus* muscles collected from WT and muscle specific *Bmal1* KO mice every 4 h from ZT0-ZT20 (Accession number: GSE43071^127^). In *mBmal1* KO mice, the region coding for the BMAL1 basic helix–loop–helix DNA binding domain was excised^127^;
3. *Gastrocnemius* (calf) muscle collected from male C57BL/6 J WT and *Clock* mutant mice every 4 h from CT22-CT46 (Accession number: GSE3746^128^). The *Clock* mutation in mice is a dominant negative (antimorphic) mutation that deletes exon 19 and causes an internal deletion of 51aa in the COOH-terminal activation domain of the clock protein^128^;
4. *Longissimus dorsi* (back) muscle collected from male C57BL/N6 mice subjected to 30 days in Bion-M1 spaceflight (microgravity; BF), Bion ground (BG) subjected to same habitat of space flown mice but exposed to gravity and flight controls in standard animal facility for the same time period. Both the space flown and the ground control mice were sacrificed on the same day within 12–14 h post-landing (Accession number: GSE94381^129^);
5. *Soleus* and *Extensor digitorum longus* muscles collected from male C57BL/N6 mice subjected to 30 days in Bion-M1 spaceflight, Bion ground subjected to same habitat of space flown mice but exposed to gravity, and flight controls in standard animal facility for the same time period. Both the space flown and the ground control mice were sacrificed on the same day within 12–14 h post-landing (Accession number: GSE80223^130^);
6. *Gastrocnemius* (calf) muscle collected from mice subjected to 11 days 19 h in STS-108 spaceflight and the controls in standard animal facility for same period. The space flown and ground control mice were sacrificed within 3.5–4 h after landing (Accession number: GSE10533^131^). In addition, different ground-based mice were subjected to 12 days hindlimb suspension or hindlimb suspension +3.5 h reloading and the controls. For more details about the microarray datasets used in this study, please refer to the Supplementary file 1.

### Acquisition of publicly available LC-MS datasets

Using a criterion of “skeletal muscle”, “mammalian tissue”, and “Proteomics” in open source repositories (PRIDE), we obtained a total of 108 samples from 3 different datasets. All three LC-MS datasets were retrieved from the mouse model system.

The LC-MS datasets were derived from:

1. *Soleus* and *Extensor digitorum longus* (*EDL*) muscles collected from male C57BL/6 J mice followed by 28 days in hypergravity (3 g) and their corresponding ground controls (1 g; Accession number: PXD017240^132^);
2. *Supraspinatus* (shoulder) muscle collected from female C57BL/6 mice subjected to 13 days in STS-135 and male C57BL/6 N mice subjected to 30 days in Bion-M1 spaceflight and their corresponding ground controls (Accession number: PXD006764^133^);
3. *Vastus lateralis*, *Soleus* and *Extensor digitorum longus* (*EDL*) muscles collected from male C57BL/6 mice subjected to 30 days in Bion-M1 spaceflight and their corresponding ground controls. Additional, *EDL* muscle tissues were collected from mice subjected to 8 days recovery post-flight and their control group (Accession number: PXD005035^134^).

For more details about the datasets used in this study, please refer to the Supplementary file 1.

### RNA-seq data analysis

For RNA-seq datasets, raw sequencing reads (fastq files) were retrieved from the NCBI-GEO and used for the pre-processing and downstream analysis. Quality assessment of raw RNA-seq reads were carried out using fastqcr R package (version 0.1.2), which helps in easy parsing and aggregation of FastQC^135^. Based on the FastQC reports, the respective adapter sequences and over-represented reads were trimmed from sequencing reads using Trimmomatic (version 0.39^136^) using default settings. After trimming, paired/single-end reads were aligned to their respective reference genome (human, mouse, and rat based on datasets). Paired/single-end reads from human datasets were aligned to human genome (*Homo sapiens* GRCh38, Ensembl release 105), from mouse datasets were aligned to mouse genome (*Mus musculus* GRCm39, Ensembl release 105), and from rat datasets were aligned to rat genome (*Rattus norvegicus* mRatBN7.2, Ensembl release 105) using STAR aligner (version 2.7.0^137^). Post-alignment, transcript-level abundances were quantified using Salmon (version 1.7.0^138^) in transcripts per million (TPM). Afterwards, tximport R package (version 1.22.0^139^) was used to import transcript-level abundance, estimate counts, and transcript lengths. After obtaining gene-level summarization from Tximport (txOut = FALSE), normalization was carried out using TMM method within edgeR package (version 3.36.0^140^). All gene features with at least 0.5 Counts per million (CPM) on average (specific to datasets) were retained and renormalized using selected features. The complete pipeline for the analysis is depicted in Fig. 1.

### Microarray data analysis

For microarray datasets, raw intensity files (CEL for Affymetrix platform and TXT for Illumina platform) were retrieved from the NCBI-GEO for the downstream analysis. Probe intensities were retrieved from raw data using limma (version 3.50.3^141^) or oligo (version 1.58.0^142^) R package based on the type of microarray datasets. Pre-processing and normalization of expression levels was carried out using Robust Multi-Array Average (RMA) procedure as implemented in limma or oligo R package. ArrayQualityMetrics R package (version 3.50.0^143^) was used for quality control and statistical testing of the arrays. No outliers were identified. The datasets were annotated based on the associated platform annotation file available on NCBI-GEO. For the set of genes that mapped to multiple probes, the probe with the highest average expression was retained.

### LC-MS data analysis

For protein datasets, raw intensity values were retrieved from the PRIDE database for the downstream analysis. MS spectra peaks detection and quantification was carried out using a standalone software MaxQuant (version 2.0.3.1^144^). It supports all main labeling techniques such as Tandem Mass Tag (TMT), as well as label-free quantification. MaxQuant performs mass calibration and database searches for protein identification, which later get quantified. Post quantification, the resulting evidence file (identified peptides and intensity values) from MaxQuant was used for downstream analysis. For the datasets (PXD017240, PXD006764, PXD005035), normalization was carried out using proteusLabelFree vignette within Proteus R package^145^.

### Rhythmicity analysis

Out of the retrieved datasets, there were three circadian RNA-seq (GSE108539, GSE109825, GSE107787) and three circadian microarray (GSE54650, GSE43071, GSE3746) datasets. Unlogged CPM values (in case of RNA-seq) and normalized expression values (in case of microarray) were used to detect 24 h rhythmic signals using RAIN R package (version 1.28.0^146^). Following multiple test correction of RAIN p-values using Benjamini-Hochberg (BH) procedure circadian (24 h rhythmic) genes were obtained using a cut-off of *q* < 0.05. The acrophase and relative amplitude were estimated for circadian gene-sets using Cosinor within Discorhythm R package (version 1.6.0^147^).

### Differential rhythmicity analysis

To detect the changes in circadian parameters (acrophase, amplitude) due to clock alterations or fasting, differentially rhythmicity analysis was carried out using Limorhyde R package^148^. After pairwise comparison (KO vs. WT) and *q* < 0.05, the list of genes that lost ~24 h rhythmicity, gained ~24 h rhythmicity, and phase-shifted (≥4 h) due to specific core-clock element deletion were obtained.

### Differential expression analysis

For the circadian RNA-seq and microarray datasets, differential expression analysis was carried out using limma within Limorhyde R package^148^. For the protein datasets, differential expression analysis was carried out using limma within Proteus R package^145^. For all other datasets, differential expression analysis was carried out directly using limma R package (version 3.50.3^141^). A cut-off of nominal *p* < 0.01 and a log fold change (FC) ≥ | 0.2| was used to obtain significantly up- or down-regulated gene sets.

### Network analysis

To gain further insight into the interactions between gene sets that were differentially expressed between spaceflight (post-37 days) and aging (80+ years vs. 20–34 years or 60–75 years vs. 20–30 years), network analysis using Cytoscape (version 3.9.1^149^) was carried out. Interactions between shortlisted genes were obtained using STRING^150^ plugin and clustered using Molecular Complex Detection plugin (MCODE^151^) within Cytoscape.

### Curation of skeletal muscle and circadian clock associated pathways

Signalling pathways associated with skeletal muscle and circadian rhythms were retrieved from the Kyoto Encyclopedia of Genes and Genomes (KEGG^66^) using KEGGREST R package (version 1.36.0^152^) and manually downloaded from the Reactome^67^ database.

Using KEGG, the retrieved pathways associated with skeletal muscle were: (1) Adipocytokine signalling (hsa04920; 69 genes) where adipocytokines play a crucial role in stimulating glucose update within skeletal muscle^153^, (2) AMP-activated protein kinase signalling (hsa04152; 120 genes) regulates glucose and lipid metabolism^154^, (3) Calcium signalling (hsa04020; 240 genes), plays a crucial role in myoblast proliferation and differentiation^155^, (4) cAMP signalling (hsa04024; 221 genes), which contributes to changes in myofibers^156^ (5) FoxO signalling (hsa04068; 131 genes), essential for disuse muscle atrophy^157^, (6) Growth hormone synthesis (hsa04935; 119 genes), crucial for muscle strength^158^, (7) Insulin resistance (hsa04931; 108 genes), characterized by elevated fasting glucose level^159^, (8) Insulin signalling (hsa04910; 137 genes), increases glucose update and metabolism within skeletal muscle, (9) Mannose type O glycan biosynthesis (hsa00515; 23 genes), important for metabolism, (10) MAPK signalling (hsa04010; 97 genes), one of the main regulators of metabolism in response to stress^160^, (11) Mineral absorption (hsa04978; 60 genes), essential for skeletal muscle mass (12) mTOR signalling (hsa04150; 155 genes), helps in the maintenance of muscle mass^161^, (13) Oxidative phosphorylation (hsa00190; 134 genes), provides primary energy source for metabolism, (14) Pi3k-Akt signalling (hsa04151; 354 genes), crucial for skeletal mass development^162^, (15) PPAR signalling (hsa03320; 75 genes), plays an important role in development, inflammation, and metabolism^163^, and (16) Vascular smooth muscle contraction (hsa04270; 134 genes), crucial for muscle contraction.

Using Reactome, the retrieved pathways associated with skeletal muscle were: (1) Calcineurin activates NFAT (R-HSA-2025928; 9 genes), crucial for skeletal muscle maintenance, (2) Cellular response to heat stress (R-HAS-3371556; 101 genes), associated with cell survival, (3) Cellular response to stress (R-HSA-2262752; 749 genes), (4) Growth hormone receptor signalling (R-HSA-982772; 24 genes), (5) Mitochondrial biogenesis (R-HSA-1592230; 96 genes), a major adaptation of skeletal muscle to exercise training^164^, (6) mTOR signalling (R-HSA-165159; 41 genes), (7) Muscle contraction (R-HSA-397014; 196 genes), (8) Myogenesis (R-HSA-525793; 29 genes), (9) Oxidative stress induced senescence (R-HSA-2559580; 95 genes), (10) Pi3K-Akt activation (R-HSA-198203; 9 genes), (11) Smooth muscle contraction (R-HSA-445355; 39 genes), and (12) Striated muscle contraction (R-HSA-390522; 36 genes).

In addition, change in skeletal mass during exercise or unloading is associated with the nuclei of muscle fibres (myonuclei^165^). Therefore, we additionally retrieved myonuclei associated genes (43 genes) from the literature^68,69^.

Using KEGG, the retrieved pathways associated with circadian rhythms were: (1) Circadian entrainment (hsa04713; 97 genes), and (2) Circadian rhythm (hsa04710; 31 genes). Using Reactome, the retrieved pathways associated with circadian rhythm was Circadian clock (R-HSA-400253; 70 genes). In addition, we included a network of circadian regulated genes (NCRG; 161 genes) previously published by our group^26^. After combining the skeletal muscle and circadian rhythms associated genes, we obtained a unique set of 2251 genes (in human) and their orthologous sets in mouse (2135 genes) and rat (2161 genes) using Biomart^166^. For the complete gene lists, please refer to the Supplementary file 2–4.

### Correlation analysis

Pearson correlation coefficient was calculated for the set of core-clock genes in spaceflight and aging datasets using the R function *cor()*. In addition, we used *rcorr()* function built in the R package Hmisc (CRAN - Package Hmisc (r-project.org)) to compute the significance levels for Pearson correlation.

## Supplementary information

Supporting Information

Dataset 1

Dataset 2

Dataset 3

Dataset 4

Dataset 5

## Data Availability

The datasets used in this study were retrieved from open-access public repositories and are available as follows: (1) Circadian RNA-seq datasets derived from human skeletal muscle (NCBI-GEO, Accession number: GSE108539, GSE109825), (2) Circadian microarray datasets derived from mouse skeletal muscle (NCBI-GEO, Accession number: GSE54650, GSE43071, GSE3746), (3) Spaceflight omics datasets derived from mouse skeletal muscle (NASA GeneLab: GLDS-99/-101/-103/-104/-105; NCBI-GEO, Accession number: GSE156321, GSE94381, GSE80223, GSE10533; PRIDE, Accession number: PXD006764, PXD005035), (4) Hypergravity associated proteomics data derived from mouse skeletal muscle (PRIDE, Accession number: PXD017240), (5) Bedrest associated microarray datasets derived from human skeletal muscle biopsies (NASA GeneLab: GLDS-370, NCBI-GEO, Accession number: GSE14798, GSE148152, GSE104999), (6) Aging associated RNA-seq datasets derived from human skeletal muscle biopsies (NCBI-GEO, Accession number: GSE97084, GSE129643), (7) Exercise associated RNA-seq datasets derived from human, mouse or rat skeletal muscle (NCBI-GEO, Accession number: GSE155230, GSE117161, GSE178262, GSE97084), and (8) Fasting associated RNA-seq dataset derived from mouse skeletal muscle (NCBI-GEO, Accession number: GSE107787).

## Code availability

All programs, packages, and functions used in this manuscript are freely available and are referred in the Methods section.
