# Supplementary material for: Skeletal muscle gene expression dysregulation in long-term spaceflights and aging is clock-dependent
Source: NPJ Microgravity. 2023 Apr 3;9:30. doi: 10.1038/s41526-023-00273-4 (PMC10070655; doi:10.1038/s41526-023-00273-4)
Supplement: Supplementary file 1 — Supporting Information [file 41526_2023_273_MOESM1_ESM.pdf]

Supporting Information

Skeletal muscle gene expression dysregulation in long-term spaceflights and aging is clock-dependent

Deeksha Malhan[1,2,3], Müge Yalçin[1,2,3], Britt Schoenrock[4], Dieter Blottner[4,5], Angela Relógio[1,2,3*]

[1]Institute for Theoretical Biology (ITB), Charité—Universitätsmedizin Berlin, Corporate Member of Freie Universität Berlin, Humboldt-Universität zu Berlin, and Berlin Institute of Health, Berlin 10117, Germany
[2]Molecular Cancer Research Center (MKFZ), Medical Department of Hematology, Oncology, and Tumour Immunology, Charité—Universitätsmedizin Berlin, Corporate Member of Freie Universität Berlin, Humboldt-Universität zu Berlin, and Berlin Institute of Health, Berlin 10117, Germany
[3]Institute for Systems Medicine and Faculty of Human Medicine, MSH Medical School Hamburg, Hamburg 20457, Germany
[4]Institute of Integrative Neuroanatomy, Charité-Universitätsmedizin Berlin, Corporate Member of Freie Universität Berlin, Humboldt-Universität zu Berlin, and Berlin Institute of Health, Berlin 10117, Germany
[5]Neuromuscular System and Neuromuscular Signaling, Center of Space Medicine Berlin, Berlin 10115, Germany

Supplementary Figures

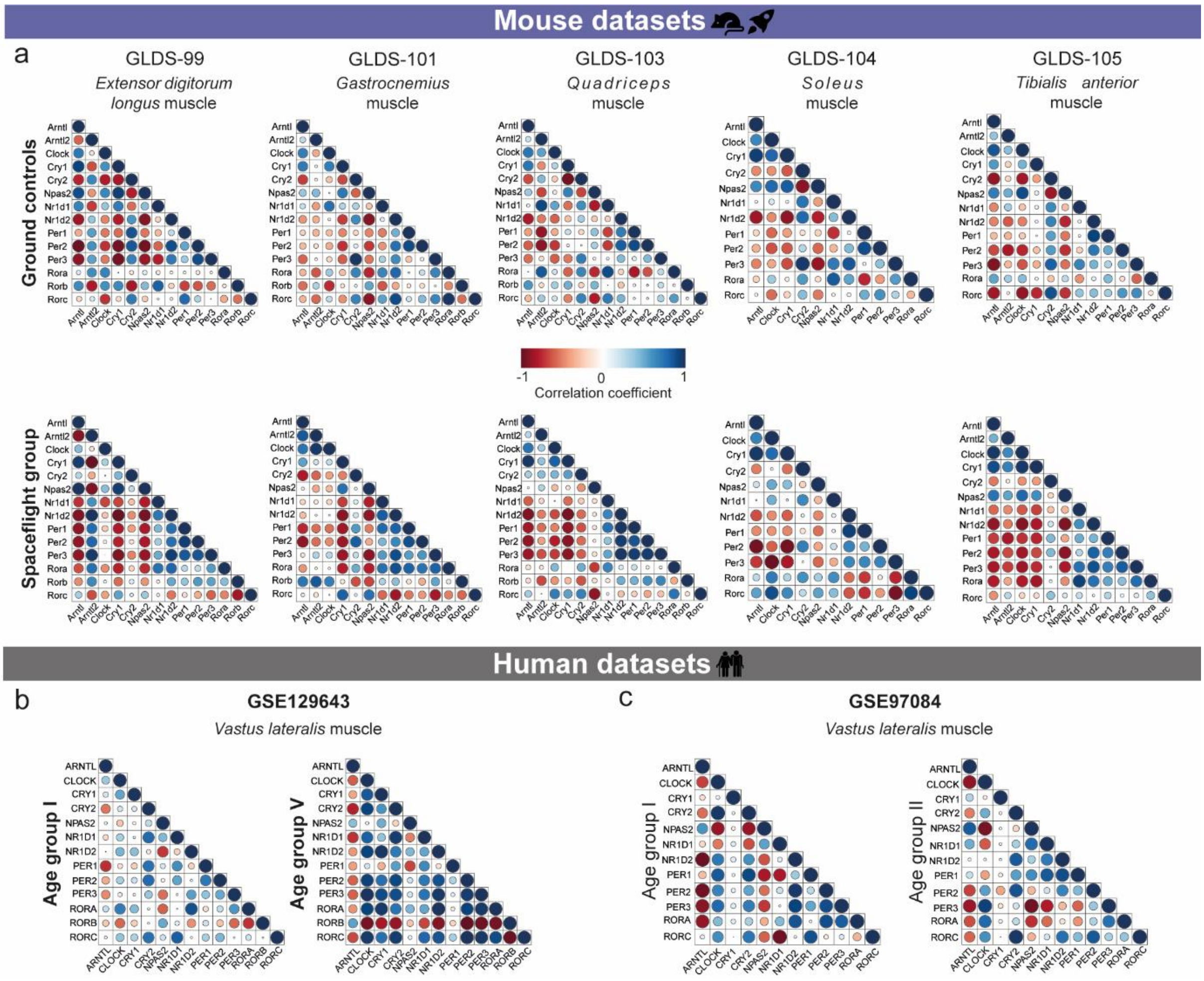

Supplementary Figure 1: Correlations plots depict the pairwise Pearson correlation of core clock genes in spaceflight and aging datasets. *a) Correlation plot in long-term spaceflight datasets (37-days), and b-c) extreme age groups from the two aging datasets b) GSE129643 and c) GSE97084. The size of the circles in the correlation plot represents the degree of correlation between specific gene pairs (large circles: strong correlation; small circles: weak correlation).*

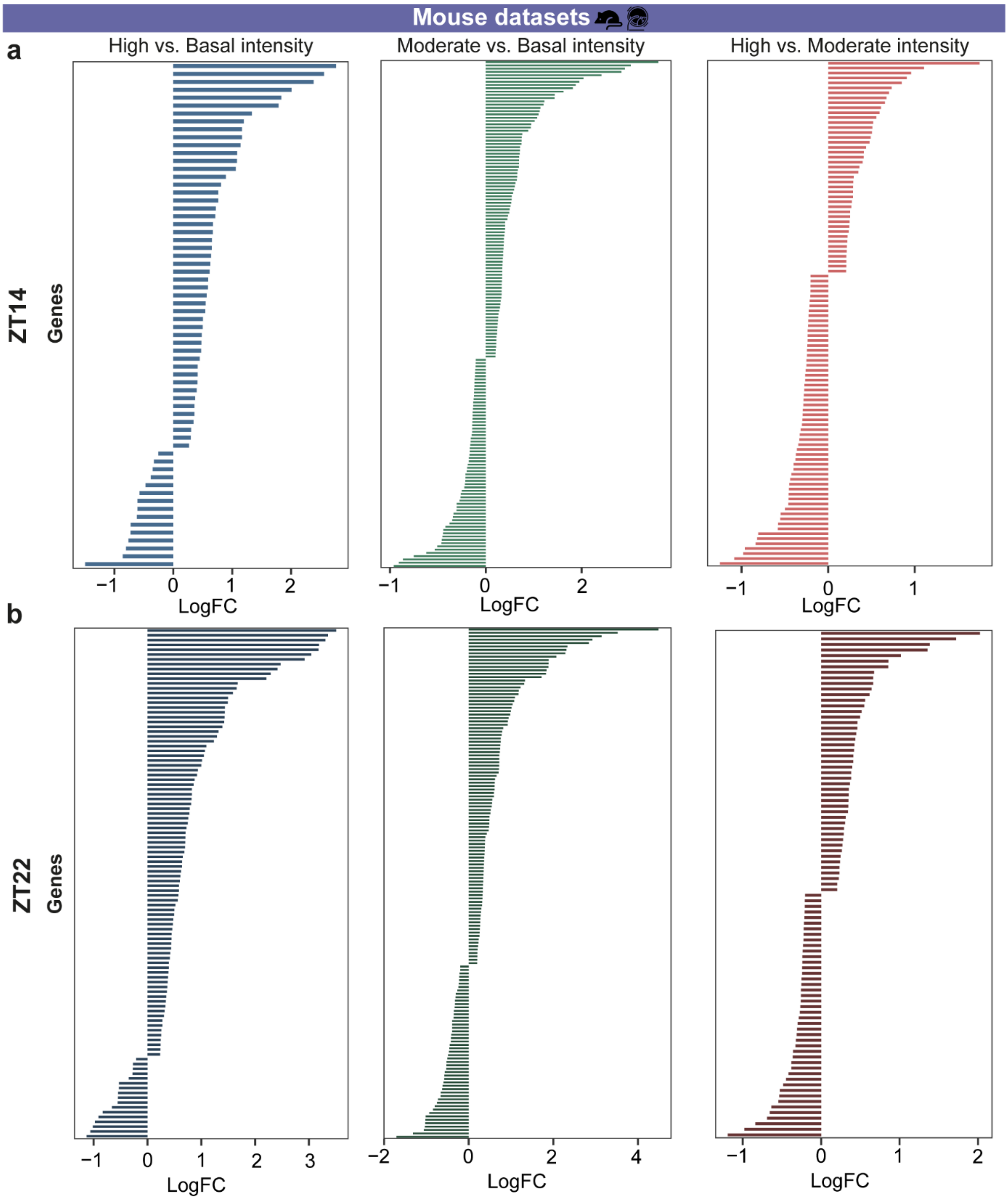

Supplementary Figure 2: Histogram depicts the up- and down-regulation among differentially expressed genes in exercise datasets retrieved from mouse skeletal muscle. *Differentially expressed genes were obtained using the criteria of logFC ≥ |0.2| and p < 0.05. Histogram depicts the distribution of differentially expressed genes identified between high vs. basal intensity (left panel), moderate vs. basal intensity (middle panel), and high vs. moderate intensity (right panel) at (a) ZT14 and at (b) ZT22.*

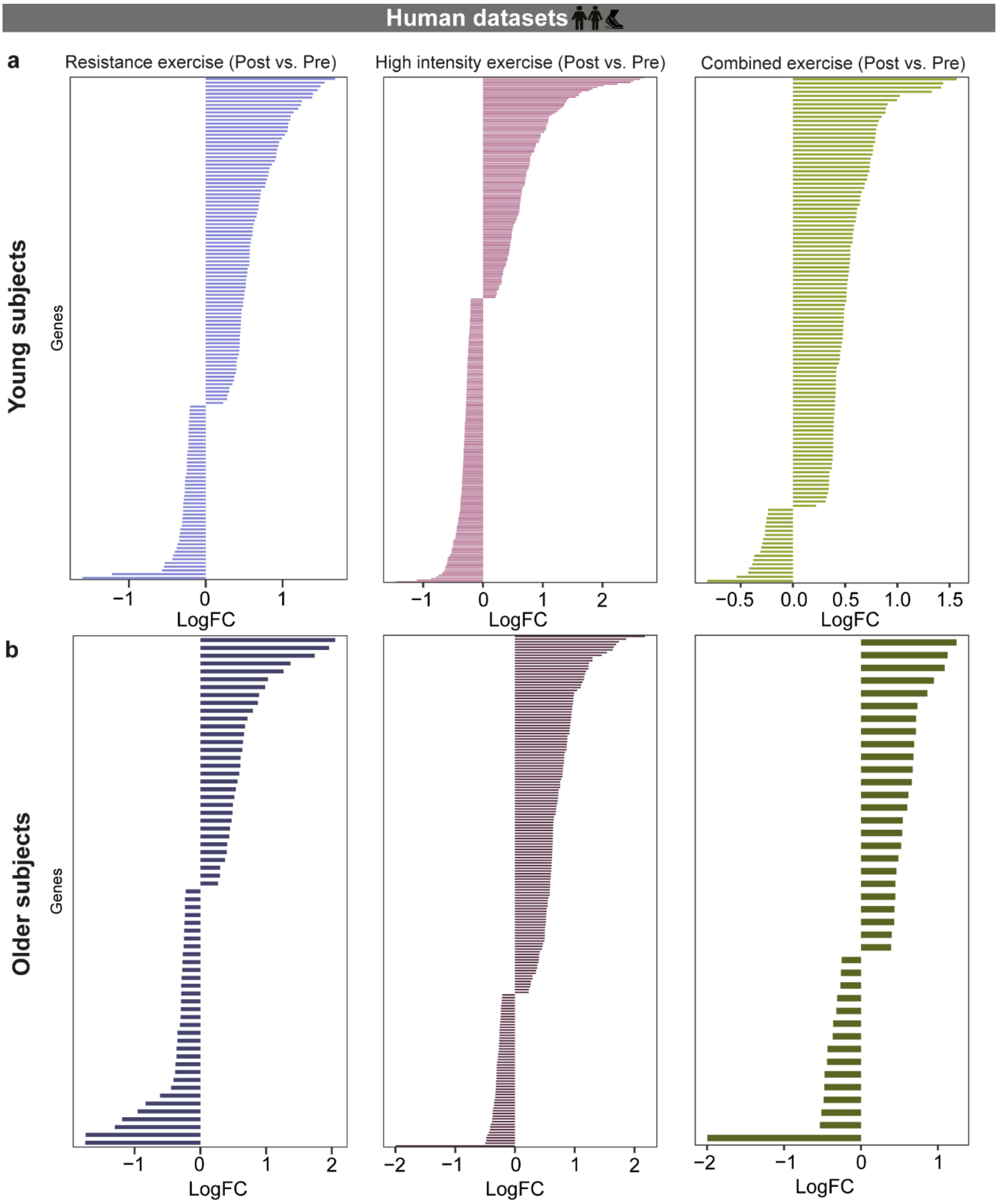

Supplementary Figure 3: Histogram depicts the up- and down-regulation among differentially expressed genes in exercise datasets retrieved from human skeletal muscle. *Differentially expressed genes were obtained using the criteria of logFC ≥ |0.2| and p < 0.05. All subjects underwent 12-weeks of exercise and their molecular profile was compared to the pre-exercise state. Histogram depicts the distribution of differentially expressed genes identified in resistance exercise (left panel), high-intensity exercise (middle panel), and combined exercise (right panel) in (a) young subjects and in (b) older subjects.*
